# Klotho: An Emerging Factor With Ergogenic Potential

**DOI:** 10.3389/fresc.2021.807123

**Published:** 2022-01-06

**Authors:** Eliott Arroyo, Ashley D. Troutman, Ranjani N. Moorthi, Keith G. Avin, Andrew R. Coggan, Kenneth Lim

**Affiliations:** ^1^Division of Nephrology & Hypertension, Department of Medicine, Indiana University School of Medicine, Indianapolis, IN, United States; ^2^Department of Physical Therapy, School of Health and Human Sciences, Indiana University Purdue University, Indianapolis, IN, United States; ^3^Department of Kinesiology, School of Health and Human Sciences, Indiana University Purdue University Indianapolis, Indianapolis, IN, United States

**Keywords:** Klotho, skeletal muscle, chronic kidney disease (CKD), sarcopenia, physical function

## Abstract

Sarcopenia and impaired cardiorespiratory fitness are commonly observed in older individuals and patients with chronic kidney disease (CKD). Declines in skeletal muscle function and aerobic capacity can progress into impaired physical function and inability to perform activities of daily living. Physical function is highly associated with important clinical outcomes such as hospitalization, functional independence, quality of life, and mortality. While lifestyle modifications such as exercise and dietary interventions have been shown to prevent and reverse declines in physical function, the utility of these treatment strategies is limited by poor widespread adoption and adherence due to a wide variety of both perceived and actual barriers to exercise. Therefore, identifying novel treatment targets to manage physical function decline is critically important. Klotho, a remarkable protein with powerful anti-aging properties has recently been investigated for its role in musculoskeletal health and physical function. Klotho is involved in several key processes that regulate skeletal muscle function, such as muscle regeneration, mitochondrial biogenesis, endothelial function, oxidative stress, and inflammation. This is particularly important for older adults and patients with CKD, which are known states of Klotho deficiency. Emerging data support the existence of Klotho-related benefits to exercise and for potential Klotho-based therapeutic interventions for the treatment of sarcopenia and its progression to physical disability. However, significant gaps in our understanding of Klotho must first be overcome before we can consider its potential ergogenic benefits. These advances will be critical to establish the optimal approach to future Klotho-based interventional trials and to determine if Klotho can regulate physical dysfunction.

## Introduction

In 1997, Kuro-o and colleagues (1) identified a novel gene that encodes a transmembrane protein with powerful anti-aging properties. The gene, Klotho, was named after Clotho—the Greek goddess who spins the thread of life and whose form is depicted in the iconic Flemish Tapestry, “The Three Fates” that hangs in the Victoria and Albert's Museum in London today. Klotho deficient mice exhibit a shortened lifespan and a premature aging syndrome that includes phenotypic features of sarcopenia and physical dysfunction such as severe muscle wasting, hypokinesis, an abnormal walking pattern, decreased stride length, muscle weakness, and decreased running endurance (1–3). Conversely, restoration of Klotho in Klotho deficient mice can reverse these aging phenotypes and extend life span (1, 4–6). In humans, several epidemiological studies have revealed a link between Klotho levels and physical function indices (7–11). Accordingly, older adults and patients with chronic kidney disease (CKD), which are known states of Klotho deficiency, commonly exhibit declines in musculoskeletal health and physical function. These observations suggest that Klotho may regulate age- and CKD-related declines in physical function.

An adequate level of physical function is essential for the performance of daily living activities and to maintain functional independence. Physical function is a cumulative response of interconnected organ systems and can be assessed by cardiorespiratory fitness, neuromuscular function (i.e., muscle strength, speed, power, and endurance), flexibility, and balance (12). The integration of these physical fitness components enables the interaction with one's environment including the ability to perform basic movements, such as walking, climbing stairs, standing up from a seated position, carrying objects, and more. These functional metrics are major determinants of whether an individual can live independently or require assistance to perform daily tasks. Importantly, poor physical function is strongly predictive of higher rates of mortality (13, 14), higher hospitalization rates (15), and poor quality of life (16). Moreover, loss of functional independence inflicts significant burden on the affected individuals and their family members and presents a major burden on healthcare economics globally. Recent estimates suggest that the United States spent $868 billion in 2015 (adjusted to 2017 medical prices) in disability-associated health care expenditures, representing 36% of total health care expenditures (17). Therefore, identifying factors associated with physical dysfunction that may be modifiable targets for interventions is critically important.

The development of physical dysfunction is complex and multifactorial. As we age, progressive declines in muscle mass, quality, and function lead to sarcopenia (18). Estimates suggest that muscle mass decreases by 1–2% per year after the age of 50, and by 3% after the age of 70 (19). Similar rates of decline have been observed in muscle strength and quality with increasing age (20, 21). Moreover, the decline in skeletal muscle power (the product of the force and velocity of muscle contraction) with advancing age occurs earlier and more rapidly than muscle strength (the ability to generate maximal muscle force) and is a stronger predictor of functional performance than muscle strength (22). The overall prevalence of sarcopenia is estimated to be between 6 and 22% in adults aged ≥ 60 years, and this prevalence increases with increasing age (23, 24), although these estimates vary widely depending on the definition, criteria, and cutoffs used to diagnose sarcopenia. In CKD, sarcopenia is driven by the accumulation of uremic toxins, inflammation, oxidative stress, ubiquitination, insulin resistance, malnutrition, aging, and sedentary lifestyle (23, 25, 26). As a result, CKD patients exhibit impaired mitochondrial function, decreased muscle quality, decreased muscle size, decreased muscle strength, and therefore a greater prevalence of sarcopenia compared to those without CKD (23, 27, 28). In addition to sarcopenia, significant declines in cardiorespiratory fitness are also observed in aging (29, 30) and CKD (31). The combination of impaired muscle function and impaired cardiorespiratory fitness ultimately reduces workload capacity and the ability to perform activities of daily living (ADLs) (12).

Beyond exercise and physical activity interventions, there are limited options for improving physical performance. In the context of sport and rehabilitation, an “ergogenic aid” refers to any pharmacological method, nutritional practice, dietary supplement, training technique, mechanical device, or psychological technique that can enhance performance and/or training adaptations (32). The term “ergogenic” comes from the Greek prefix *ergo-* meaning “work” and the suffix *-gen* meaning “production.” As such, any intervention or therapeutic agent that enhances or restores the ability to produce work can be described as “ergogenic.” Accumulating data suggest that Klotho may play a key role in the development of sarcopenia and its progression into physical dysfunction. Moreover, research in animal models suggests that enhancing Klotho levels may improve skeletal muscle strength, quality, and recovery following injury and may therefore represent a novel ergogenic agent (5, 33, 34).

This comprehensive review considers the biological mechanisms of Klotho, detrimental effects imposed by its deficiency on physical function in aging and CKD, and explores contemporary evidence supporting the potential ergogenic benefits of therapeutic targeting of Klotho.

## Sarcopenia in Aging and CKD: an Opportunity for Klotho

In 1989, Dr. Irwin H. Rosenberg first coined the term sarcopenia (from the Greek: *sarx* for flesh and *penia* for loss) to describe the striking age-related declines observed in muscle mass and muscle function (35). In 2016, sarcopenia became recognized as an independent condition with an International Classification of Disease, Tenth Revision, Clinical Modification (ICD-10-CM) code M62.84 (36). While some argue that the term “sarcopenia” should be exclusive to older persons (≥ 60 years of age) (37), consensus definitions proposed by expert panels recognize sarcopenia as a muscle disease that can develop early in life with a complex, multifactorial etiology (38–43). Unhealthy lifestyle choices and the presence of chronic diseases are capable of accelerating muscle loss in middle-aged and older adults (44). Notably, the European Working Group on Sarcopenia in Older People (EWGSOP) makes a distinction between “primary” or age-related sarcopenia and “secondary” sarcopenia (40, 41). Primary sarcopenia is when the etiology is related to aging alone, while secondary sarcopenia results from other conditions that can be concomitant or not with aging and that can occur early in the adult life, such as low levels of physical activity, systemic disease, and malnutrition. In the field of nephrology, other terms have been used to describe changes in skeletal muscle with CKD, including protein-energy wasting, cachexia, and frailty. While these terms have distinct definitions from sarcopenia (25), they share common criteria and clinical outcomes. For the purposes of this review, the condition of skeletal muscle loss and dysfunction in aging and CKD will be referred to as sarcopenia.

Changes in skeletal muscle during aging and CKD can progress into muscle weakness, decreased mobility, increased fatigue, increased risk of metabolic disorders, and increased risk of falls and skeletal fractures. With aging, there is a progressive loss of motor units, type II muscle fiber atrophy, and a net replacement of fast (type II) motor units by slow (type I) motor units (18, 45). These neuromuscular changes result in the progressive loss of muscle power, i.e., the product of force and speed, which is necessary for daily tasks, such as climbing steps, rising from a chair, or avoiding falls and resultant injury after a perturbation in balance (46). Muscle quality also declines with increasing age, with mitochondrial dysfunction promoting intramuscular fat deposition (47). Older men have considerably more fat and connective tissue within the muscle compartments of the thigh than younger men (48), and a previous study showed a yearly increase of 18% in intramuscular adipose tissue in older adults (49). Intramuscular lipid infiltration is associated with muscle weakness and poor physical function (50, 51), independent of muscle size. Additionally, aging is associated with a negative protein balance caused by decreased expression of anabolic factors and increased expression of catabolic endocrine and inflammatory factors (18). The capacity for skeletal muscle regeneration also declines with age due to reduced muscle innervation, increased fibrosis, altered expression of growth factors and cytokines, reduced number and altered proliferative potential of satellite cells, telomere shortening in satellite cells, and enhanced apoptosis (52). Clearly, a wide variety of factors play a role in skeletal muscle deterioration. While these factors are observed during “normal” aging, they overlap with factors that contribute to sarcopenia in CKD.

The relationship between kidney function and aging is well-recognized and appears bidirectional. The kidneys are among the organs that are most affected by aging (53), and the uremic phenotype in CKD is characterized by many conditions associated with aging, such as osteoporosis, atherosclerosis, inflammation, oxidative stress, insulin resistance, skin atrophy, and cognitive dysfunction (54, 55). Similarly, the accumulation of uremic toxins in CKD leads to the premature deterioration of muscle function that resembles “primary” sarcopenia (25). Muscle loss is a frequent finding in CKD, especially for patients with more advanced stages of CKD undergoing hemodialysis (56–58). These musculoskeletal deficits are linked to worse quality of life, sedentary behavior, fracture risk, cardiovascular complications, graft failure, transplant post-operative complications, increased hospitalization, and mortality (59–62). CKD patients exhibit 50 to 120% lower scores in physical performance compared to non-uremic reference controls (12) and CKD severity is associated with worse physical performance (63, 64). One study found that scores on the Short Physical Performance Battery (SPPB) test were 0.51 points lower for CKD Stage 3; 0.61 points lower for CKD Stage 4; and 1.75 points lower for CKD Stage 5 compared to the SPPB scores of patients with an estimated glomerular filtration rate (eGFR) >60 ml/min/1.73 m^2^ (63). Even relatively high-functioning advanced CKD (Stages 4–5) patients have been shown to have significantly lower levels of physical function than older adults with heart failure and chronic obstructive pulmonary disease (65). A recent study using a large cohort from the United Kingdom found that one in 10 participants with CKD (eGFR <60 ml/min/1.73 m^2^) had probable sarcopenia (defined as low grip strength), almost twice that observed in those without CKD (23). Therefore, CKD patients represent a particularly vulnerable population with respect to sarcopenia.

Evidence suggests that Klotho levels are reduced as early as stage 2 CKD (66) and progressively decrease with advancing CKD stage (67). Similarly, Klotho levels have been shown to decline with increasing age (68). Over the past decade, studies in Klotho deficient mice (3) and older adults (7–11) have revealed a strong association between Klotho levels and skeletal muscle strength and physical function. Therefore, poor physical function in older adults and CKD patients may represent a unique opportunity for Klotho-based therapy. In the next sections, the biology of Klotho and its role in skeletal muscle will be discussed.

## Klotho: Biology, Tissue Expression, and Functions

The human *Klotho* gene consists of five exons and four introns, located in chromosome 13q12 with a size of 50 kb (69). The gene encodes a type 1 transmembrane glycoprotein consisting of 1,012 amino acids with a molecular weight of 135 kDa (1, 70). Full-length Klotho consists of a putative N-terminal signal sequence and two internal repeats (KL1 and KL2) constituting the extracellular domain, a single-pass transmembrane domain, and a short intracellular cytosolic domain of 10 amino acids at the C-terminus (69, 71). A soluble isoform of Klotho (sKlotho) also exists in circulation generated from the cleavage of full-length Klotho in an α-cut by the membrane proteases A Disintegrin and Metalloproteinase (ADAM)10 and ADAM17 (72). This process generates a 130 kDa soluble protein that contains both extracellular domains (KL1 and KL2) but lacks the transmembrane and intracellular components. A smaller 65 kDa isoform consisting of only the KL1 domain can also generated by a ß-cut (72). Little is known regarding the distinct roles of each of these isoforms. Soluble Klotho is present in blood, urine, and cerebrospinal fluid (73, 74).

Klotho is highly expressed in the kidney, mainly in the distal tubules and, to a lesser extent, in the proximal tubules (1, 75). Elimination of renal Klotho expression using a novel mouse strain with Klotho deleted throughout the nephron was shown to reduce circulating levels by ~80% (76), revealing that the kidney is the main source of circulating Klotho. Similarly, unilateral nephrectomy in humans significantly reduces circulating levels of Klotho (77). Moreover, a recent meta-analysis found that there is a significant reduction in serum Klotho levels after a living kidney donation and an increase in serum Klotho levels after receiving a successful kidney transplant (78). The kidney has dual roles in regulating Klotho homeostasis by both producing and releasing Klotho into circulation and by clearing sKlotho from the blood into the urinary lumen (75).

Klotho is also expressed in other tissues; however, extra-renal expression of Klotho appears to be less abundant than expression at the kidneys (1, 79). In a recent review article, Olauson and colleagues (80) compiled data from publicly available RNA-Seq and mass spectrometry databases (The FANTOM5 project, The Human Protein Atlas, The GTEx Consortium and Illumina Body Map, and The Human Proteome Map) to propose a classification for Klotho expression in tissues based on current evidence for basal mRNA and protein expression levels. Tissues with high Klotho expression include: distal tubules, parathyroid gland, sinoatrial node and choroid plexus; tissues with intermediate expression include: proximal tubules, brain, eye, inner ear, anterior pituitary, pancreas, lung, testis, ovaries, and placenta; and tissues with low or absent expression include: skeletal muscle, bone, cartilage, skin, adipose tissue, liver, spleen, heart, arteries, blood and immune cells, and parts of the gastrointestinal tract (80). Although skeletal muscle expression of Klotho is reportedly low, limited evidence suggests that Klotho transcript and protein levels are elevated in response to injury (34, 81, 82). The role of endogenous Klotho in skeletal muscle regeneration is discussed in a later section.

A major function for Klotho is serving as an obligate co-receptor for fibroblast growth factor 23 (FGF23) (83). FGF23 is a hormone produced by osteocytes that promotes urinary phosphate excretion and lowers serum levels of 1,25-dihydroxyvitamin D_3_ (1,25(OH)_2_D_3_), the active hormonal form of vitamin D (84). FGF23-null (*FGF23*^−/−^) mice were shown to develop hyperphosphatemia and high serum 1,25(OH)_2_D_3_, as well as premature aging features identical to *Klotho*-knockout (*KL*^−/−^) mice (2, 85). FGF23 signaling requires Klotho to bind to its receptors (FGFR) with high affinity, and the Klotho-FGFR complex functions as the physiological receptor for FGF23 (83). Structural studies revealed that Klotho extends a receptor-binding arm which interacts directly with the FGFR1c isoform, forming a groove for the FGF23 ligand (71). FGF23 signaling *via* the Klotho–FGFR complex regulates the function of the kidneys and the parathyroid glands to regulate phosphate homeostasis.

Both membrane-bound and sKlotho associate with cognate FGFR isoforms FGFR1c, FGFR3c, and FGFR4, enabling them to bind and respond to FGF23 (83, 86, 87). Binding of FGFRs expressed by the kidneys stimulates urinary phosphate excretion by inducing the internalization and degradation of sodium-dependent phosphate transport protein 2A (NPT2A) and downregulating the expression of *NPT2A* (also known as *SLC34A1*). *NPT2A* downregulation results in decreased phosphate reabsorption in renal proximal tubules and increased urinary phosphate excretion (88). Systemic regulation of phosphate homeostasis *via* FGF23 is dependent on Klotho, and this is reflected by one study demonstrating no systemic effects on phosphate balance following administration of recombinant bioactive FGF23 into *KL*^−/−^ mice, *in vivo* (2).

Activation of the main circulating form of vitamin D_3_, 25-hydroxyvitamin D (25-OH-D_3_), to its active form, 1,25(OH)_2_D_3_, occurs in the kidney and involves a series of cytochrome P450-containing sterol hydroxylases to generate and degrade the active hormone (89, 90). FGF23 suppresses the expression of *CYP27B1*, which encodes 25-hydroxyvitamin D_3_ 1α hydroxylase, the enzyme that converts 25-OH-D_3_ to 1,25(OH)_2_D_3_ (91). FGF23 also increases the expression of *CYP24AL* which encodes 24-hydroxylase, the key enzyme responsible for vitamin D catabolism (89). Therefore, mice lacking FGF23 or Klotho exhibit phosphate retention due to impaired urinary phosphate excretion and vitamin D intoxication due to increased synthesis and decreased degradation of 1,25(OH)_2_D_3_.

The soluble form of Klotho can exert a wide variety of extra-renal actions that are independent of FGF23. Circulating Klotho has been shown to regulate the activity of several signaling pathways including insulin-like growth factor (IGF)-1 (6, 92), cyclic adenosine monophosphate (93), Protein kinase C (94), p53/p21 (95), Wnt (82, 96–98), and transforming growth factor(TGF)β1 (99). Additionally, Klotho has been shown to inhibit oxidative stress (100, 101) and inflammation (102). Mounting evidence suggests that Klotho can also exert cardio-protective (103) and vasculo-protective effects (104), which is discussed in greater detail in a later section. In summary, sKlotho regulates multiple signaling pathways to exert pleiotropic functions with far reaching implications on health and longevity. The potential role of Klotho in regulating musculoskeletal health has only recently been explored and will be discussed in further detail next.

## Klotho and Skeletal Muscle

The current approaches for the treatment and prevention of sarcopenia focus on exercise prescription and dietary interventions, which have promoted favorable outcomes in both aging adults (105) and CKD (106). However, the widespread adoption of exercise programs and physical activity recommendations by older adults (107) and patients with CKD (108) has been poor due to a wide range of barriers such as time availability, lack of resources, co-morbidities, lack of transportation, and post-dialysis fatigue in the case of dialysis patients. Therefore, a comprehensive approach that also incorporates pharmacological interventions may be a more effective strategy to combat sarcopenia. Advances in our understanding of the pathophysiology of sarcopenia have provided potential targets for drug discovery. Pharmacological strategies for the treatment of muscle loss include testosterone therapy (109–111), growth hormone (112), myostatin inhibitors (113), activin receptor antibodies (114), metformin (115), the angiotensin-converting enzyme inhibitor, perindopril (116), the ghrelin agonist, anamorelin (117), and sarconeos (BIOL101), a novel drug containing 20-hydroxyecdysone, which activates the MAS (angiotensin-1) receptor in muscle cells (118). Notably, the anti-aging effects of Klotho have been partially attributed to modulation of the renin-angiotensin system (RAS), Wnt, mammalian target of rapamycin (mTOR), and mitogen-activated protein kinase (MAPK) pathways (92, 96, 119, 120), some of which are also mechanistic targets for these sarcopenia treatments. Additionally, testosterone administration was shown to upregulate renal Klotho mRNA expression (121). These observations suggest a potential mechanistic link between sarcopenia and Klotho levels.

Data from the Invecchiare in Chianti (Aging in the Chianti Area; InCHIANTI) study, a population-based longitudinal study of older adults in Italy, revealed that an increase in plasma Klotho was independently associated with higher grip strength (ß = 1.20, SE = 0.35, *p* < 0.001) in participants with plasma Klotho <681 pg/mL (8). In the same cohort, higher plasma Klotho was also independently associated with lower risk of ADL disability (OR = 0.57, 95% CI 0.35–0.93, *p* = 0.02) (7), higher average Short Physical Performance Battery (SPPB) scores (ß = 0.49, 95% CI 0.08–0.86, *p* = 0.02) (11), and lower risk of frailty (OR = 0.46, 95% CI 0.21–0.98, *p* = 0.045) (10). Similarly, data from the Health, Aging, and Body Composition (Health ABC) study showed that older adults in the highest tertile of plasma Klotho (>747 pg/mL) exhibited higher knee extension strength compared with those in the lowest tertile (<536 pg/mL; β = 0.72, SE = 0.018, *p* < 0.0001) and a lower decline in knee strength over 4 years of follow-up (β = −0.025, SE = 0.011, *p* = 0.02) (9). More recently, data from the Physical Fitness as Klotho Protein Stimulator; Antiaging Effects of New Training Methods (FIT-AGING) study, a randomized controlled trial conducted in Spain, found significant positive relationships between sKlotho and handgrip strength, knee extension peak torque, and lean mass index in middle-aged adults (122, 123). In a small cohort of dialysis patients, patients with lower plasma sKlotho (< 369 pg/mL) presented a higher risk of having low handgrip strength and low performance on the 6-min walk test and the sit-to-stand test (124). Observational studies evaluating the relationship between Klotho levels and indices of physical function and health are summarized in Table 1. These clinical observations are consistent with experimental data: Phelps and colleagues (3) found that maximum forelimb grip force measured in Klotho deficient mice were 53% and 51% lower than wildtype and Klotho overexpressing (*EFmKL46*) mice, respectively. Moreover, Klotho deficient mice exhibited an endurance capacity ~60% less than both wildtype and *EFmKL46* mice. Another study found a reduction in the number of muscle stem cells and smaller muscle size in Klotho deficient mice compared to control mice at the age of 8 weeks (33). The reduction in muscle mass in Klotho deficient mice was concomitant with the increased muscle expression of ubiquitin ligases, suggesting that Klotho deficient mice are likely undergoing sarcopenia-like muscle wasting rather than a developmental defect in skeletal muscle growth. Several potential biological mechanisms may explain the role of Klotho on regulating skeletal muscle function and this is discussed in the next section.

**Table 1:** Relationship between Klotho levels and indices of physical function and health.

**Reference**	**Study design**	**Population**	**Sample and technique**	**Key findings**
Amaro-Gahete et al. (123)	Cross-sectional study in the FIT-AGING study cohort.	Middle-aged sedentary adults (*n* = 74, 35 men, age 53.7 ± 5.1 years)	-Plasma -ELISA (Demeditec, Kiel, Germany)	-sKlotho is positively associated with lean mass index after controlling for age and sex.
Amaro-Gahete et al. (122)	Cross-sectional study in the FIT-AGING study cohort.	Middle-aged sedentary adults (*n* = 74, 35 men, age 53.7 ± 5.1 years)	-Plasma -ELISA (Demeditec, Kiel, Germany)	-sKlotho is positively associated with VO_2_max, knee extension peak torque, and hand grip strength.
Baldan et al. (125)	Cross-sectional study.	β-thalassemia major (ß-TM) patients (*n* = 106, 43 men, age 38.6 ± 6.5 years) and healthy blood donors (*n* = 95, 70 men, age 40.9 ± 7.8 years)	-Plasma -ELISA (IBL Ltd., Takasaki, Japan)	-sKlotho levels are lower in patients with ß-TM compared to healthy controls. -Up to an estimated sKlotho's threshold value of 580 ± 149 pg/mL, a weak linear correlation was observed between sKlotho and normalized hand-grip strength in ß-TM patients. -No correlation was found for Klotho values above 580 pg/mL.
Chalhoub et al. (126)	-Prospective study in the Health, Aging, and Body Composition (Health ABC) study cohort. -Patients were followed up for a median of 5 years.	Well-functioning older adults (*n* = 2,776, aged 70 to 79 years) were divided into sKlotho quartiles: quartile 1: 320.6–437.3 pg/mL; quartile 2: 521.6–592.1 pg/mL; quartile 3: 670.1–756.2 pg/mL; and quartile 4: 887.7–1186.4 pg/mL.	-Serum -ELISA (IBL Ltd., Takasaki, Japan)	-No differences were observed between quartiles on gait speed, grip strength, or appendicular lean mass. -sKlotho levels are not associated with bone mineral density, bone loss, or fracture risk in older individuals.
Crasto et al. (7)	Cross-sectional study in the Invecchiare in Chianti (InCHIANTI) study cohort.	Population-based sample of 802 adults aged 65 years and older divided into sKlotho tertiles: tertile 1: <575 pg/mL; tertile 2: 575–763 pg/mL; and tertile 3: >763 pg/mL	-Plasma -ELISA (IBL Ltd., Takasaki, Japan)	-Lower levels of sKlotho were associated with older age, lower SPPB score, lower cognitive function, and higher ADL disability. -Low sKlotho was associated with ADL disability after adjusting for age, education, SPPB, congestive heart failure, and diabetes.
Dote-Montero et al. (127)	Cross-sectional study in the FIT-AGING study cohort.	Middle-aged sedentary adults (*n* = 73, 34 men, age 53.8 ± 5.1 years)	-Plasma -ELISA (Demeditec, Kiel, Germany)	-sKlotho was positively associated with DHEAS in men but not women, but this disappeared after adjusting for age. -sKlotho was positively associated with testosterone in both men and women. -The association between sKlotho and testosterone disappeared after adjusting for age in men, but not in women.
Fukasawa et al. (128)	Cross-sectional study.	Hemodialysis patients (*n* = 77, 52 men, median age = 67.0 years, IQR = 60.0–73.0 years)	-Plasma -ELISA (IBL Ltd., Takasaki, Japan)	No significant correlation between sKlotho levels and abdominal muscle area or creatinine production.
Matsubara et al. (129)	Cross-sectional study.	Healthy and postmenopausal women (*n* = 69, aged 60 ± 1)	-Plasma -ELISA (IBL Ltd., Tokyo, Japan)	-sKlotho and carotid arterial compliance were positively correlated after adjusting for age, pulse pressure, and BMI. -sKlotho is positively correlated with VO_2_ at VT after adjusting for age and BMI.
Mostafidi et al. (130)	-Cross-sectional study. -Blood samples were obtained after an 8 hour fast the morning following an afternoon training session.	Healthy male football players (*n* = 30, aged 18–22 years) who were performing daily morning and evening exercise training and healthy non-athlete male controls (*n* = 28, age 18–27 years).	-Plasma -ELISA (Hangzhou Eastgbiopharm Co., Ltd., Hangzhou, China)	-sKlotho levels are significantly higher in football players compared to healthy controls with normal physical activity levels.
Patel et al. (131)	Cross-sectional study.	Never smokers (*n* = 13, 10 men, age 65 ± 8), smokers with normal spirometry (*n* = 13, 5 men, age 51 ± 7), and COPD patients (*n* = 61, 38 men, age 64 ± 10).	-Vastus lateralis protein expression and serum. -ELISA (IBL Ltd., Japan) -Immunohistochemistry (rabbit anti-Klotho antibody [diluted 1:100], Aviva Systems Biology, San Diego, CA, USA)	-Quadriceps Klotho levels were lower in smokers compared to non-smokers. -Quadriceps Klotho levels were higher in patients with low fat free mass index and low quadriceps strength. -sKlotho had an independent association with quadriceps strength but did not relate to quadriceps Klotho levels. -Klotho protein was localized to the muscle fiber membrane and associated with centralized nuclei.
Patel et al. (131)	Mice exposed to air or 4% cigarette smoke for 2 hours/day, 5 days/week, for 77 weeks were evaluated for Klotho expression in the context of muscle damage induced by electroporation.	Female C57BL/6 mice, 3–4 months of age divided into sham (*n* = 9) or cigarette smoke (*n* = 19).	Gastrocnemius protein expression -ELISA (IBL Ltd., Japan) -Immunohistochemistry (rabbit anti-Klotho antibody [diluted 1:100], Aviva Systems Biology, San Diego, CA, USA)	-Significant Klotho protein expression present in damaged skeletal muscle tissue, but not healthy tissue. -Klotho co-localized to both the plasma membrane and to centralized nuclei. -↓ in gastrocnemius Klotho protein levels in mice exposed to smoke compared to sham.
Phelps et al. (3)	Klotho deficient mice were compared to transgenic Klotho overexpressing mice and wildtype mice for strength and running endurance.	Klotho deficient mice (Klotho homozygous males, C57BL/6), transgenic Klotho overexpressing mice (EFmKL46, homozygous, males and females, C57BL/6), and wildtype mice.	-Kidney and Gastrocnemius mRNA expression -RT-qPCR analysis	-Gastrocnemius Klotho mRNA expression level in EFmKL46 mice was 70-fold higher compared to wildtype mice. -Klotho expression was not detected in the gastrocnemius of Klotho deficient mice. -Forelimb grip force 53% and 51% lower in Klotho deficient mice compared to wildtype and EFmKL46 mice, respectively. -Klotho deficient mice spent 66% and 62% less time running than wildtype and EFmKL46 mice, respectively.
Polat et al. (132)	Cross-sectional study.	Frail (*n* = 45, 14 men, age 79.4 ± 6.9 years) and non-frail (*n* = 44, 17 men, age 72.7 ± 4.5 years) older adults.	-Serum -ELISA (IBL Ltd., Takasaki, Japan)	-No significant difference in sKlotho between frail and non-frail patients. -No significant correlation between sKlotho and frail score.
Rosa et al. (133)	Cross-sectional study.	Master endurance runners (*n* = 18, age 53 ± 8.2 years), master sprinters (*n* = 13, age 50 ± 8.9 years), untrained healthy young (*n* = 17, age 22.7 ± 3.9 years), and untrained middle-aged (*n* = 12, age 45.5 ± 9.8 years) men.	-Serum -ELISA (IBL Ltd., Japan)	-sKlotho levels were higher in master sprinters than master endurance runners and untrained middle-aged men. -sKlotho levels were similar between young untrained men and master sprinters. -sKlotho levels were higher in young untrained men than master endurance runners and untrained middle-aged men.
Sanz et al. (134)	-Prospective study. -Patients were followed up for 6 months.	Older adults living in nursing homes in Gipuzkoa, Spain (*n* = 103, 30 men, age 84.7 ± 6.96 years)	-Serum -ELISA (IBL Ltd., Gunma, Japan)	-Low sKlotho levels were associated with a lower score in the psychological component of the Tilburg Frailty Indicator, greater dependence in activities of daily living, and more falls during the 6-month follow-up.
Semba et al. (8)	-Prospective study in the Invecchiare in Chianti (InCHIANTI) study cohort. -Patients were followed up for 6 years.	Population-based sample of 804 adults aged 65 years and older.	-Plasma -ELISA (IBL Ltd., Takasaki, Japan)	-sKlotho was positively associated with grip strength at a threshold of <681 pg/mL after adjusting for age, sex, education, smoking, physical activity, cognition, and chronic diseases.
Semba et al. (9)	-Prospective study in the Health, Aging, and Body Composition (Health ABC) study cohort. -Patients were followed up for 4 years.	Well-functioning older adults (*n* = 2,734, aged 71 to 80 years) were divided into sKlotho tertiles: tertile 1: <536 pg/mL; tertile 2: 536–747 pg/mL; and tertile 3: >747 pg/mL.	-Plasma -ELISA (IBL Ltd., Takasaki, Japan)	-The highest tertile of sKlotho had higher knee extension strength compared with those in the lowest tertile after adjusting for age, sex, race, smoking, study site, inflammatory markers, and diabetes. -The highest tertile of sKlotho had less of a decline in knee strength over 4 years of follow-up compared with those in the lowest tertile after adjusting for the same covariates above.
Shardell et al. (11)	-Prospective study in the Invecchiare in Chianti (InCHIANTI) study cohort. -Patients were followed up for 6 years.	Population-based sample of 860 adults aged 55 years and older split by sKlotho median (669 pg/mL)	-Plasma -ELISA (IBL Ltd., Takasaki, Japan)	-Higher sKlotho levels were associated with higher average SPPB scores after adjustment for covariates.
Shardell et al. (10)	-Prospective study in the Invecchiare in Chianti (InCHIANTI) study cohort. -Patients were followed up for 3 years.	Population-based sample of 774 adults aged 65 years and older split by sKlotho median (660 pg/mL)	-Plasma -ELISA (IBL Ltd., Takasaki, Japan)	-Higher sKlotho levels were associated with lower odds of frailty after adjustment for covariates. -Higher sKlotho was particularly associated with lower odds of exhaustion.
Valenzuela et al. (124)	-Prospective study in an elderly dialysis cohort. -All-cause mortality was registered 18 months later.	ESRD male dialysis patients (*n* = 30, age 71 ± 9 years) split by sKlotho median (369 pg/mL)	-Plasma -ELISA (IBL Ltd., Japan)	Lower sKlotho levels were associated with higher risk of having low handgrip strength and low performance on the 6-min walk test and the sit-to-stand test.

*ELISA, enzyme-linked immunosorbent assay; sKlotho, soluble Klotho; VO_2_max, maximal oxygen uptake; IBL, Immuno-Biological Laboratories; ß-TM, β-thalassemia major; SPPB, short physical performance battery; ADL, activities of daily living; DHEAS, dehydroepiandrosterone sulfate; BMI, body mass index; VO_2_ at VT, oxygen consumption at the point of ventilatory threshold; RT-qPCR, reverse transcription quantitative polymerase chain reaction*.

### Klotho and Muscle Regeneration

Skeletal muscle is capable of full regeneration and recovery following even the most extreme damaging events, including crush injuries, freezing injuries, injection of toxins, and exercise-induced damage (i.e., eccentric muscle contractions). Recovery of normal structure and function following muscle damage relies largely on the functions of mononucleated, myogenic stem cells, called satellite cells, that retain their ability to proliferate and then differentiate to fuse with existing fibers or with other myogenic cells to generate new fibers (135). Muscle regeneration is an organized process involving a local inflammatory response, followed by the activation, proliferation, and differentiation of quiescent satellite cells, culminating in remodeling and regeneration of damaged tissue (135, 136). Tight regulation of satellite cell differentiation and their self-renewal is essential during this process to prevent depletion of the satellite cell pool and facilitate sufficient myoblasts for myogenesis. Numerous factors can influence the muscle regenerative response, and perturbations in satellite cell function can lead to loss of muscle function and negative health outcomes. Moreover, muscle regeneration is compromised in several conditions by excessive deposition of extracellular matrix (ECM), resulting in muscle fibrosis (137). The regenerative potential of skeletal muscle has been shown to diminish with advanced age and in CKD (138, 139).

Recent studies have shown that Klotho plays a key role in regulating muscle regeneration. Ahrens and colleagues (33) found that Klotho deficient mice showed evidence of muscle atrophy, fibrosis, calcification, and scarring at 10 and 21 days following cardiotoxin-induced injury of skeletal muscle (50 μL cardiotoxin injected into the tibialis anterior muscle). Furthermore, differentiation of myofibers and muscle satellite cell self-renewal and function were severely compromised. Similarly, Sahu et al. (34) demonstrated that Klotho deficient mice exhibited a decreased regenerative index, smaller myofiber size, and increased fibrosis when compared to wild-type mice following skeletal muscle injury. A study in *mdx* mice, the mouse model for Duchenne muscular dystrophy (DMD), showed that that epigenetic silencing of *Klotho* in muscle occurs at the onset of DMD and contributes to the disturbed muscle regeneration and muscle fibrosis observed in *mdx* mice (140). Interestingly, *Klotho* gene silencing in *mdx* mice does not affect levels of sKlotho or expression in brain, kidney, pancreas, or spleen. This suggests that Klotho-mediated effects on skeletal muscle homeostasis may be largely paracrine or autocrine, and endocrine effects may not be sufficient to replace loss of locally expressed Klotho in muscle in the context of dystrophin deficiency. Therefore, Klotho replacement therapy may not be an effective treatment strategy for DMD.

Evidence suggests that changes in local expression of Klotho within injured muscle is critical for regeneration of functional myofibers. Following cardiotoxin-induced muscle injury in young and aged male mice, Klotho is highly upregulated at both the mRNA and protein level 14 days following injury within the regenerating sites of young skeletal muscle, but not in aged muscles with impaired regeneration (34). Circulating Klotho levels followed a similar expression pattern. Moreover, muscle satellite cells were shown to express Klotho protein and secrete sKlotho, though both expression of Klotho and sKlotho secretion were blunted in aged muscle. To evaluate the contribution of endogenous Klotho in functional muscle regeneration, Sahu et al. (34) injected the tibialis anterior of young mice with GFP-tagged SMARTpool® lentiviral particles carrying shRNA to Klotho before inducing injury. Muscles treated with shRNA to Klotho showed a significant decrease in Klotho protein expression at the site of injury as well as decreased circulating Klotho. This suggests that muscle-derived Klotho may contribute to the increase observed in circulating Klotho levels after injury. Welc et al. (82) showed that *Klotho* gene expression is reduced by ~69% at 3 days post-injury, followed by a 385% increase at 7 days post-injury compared to 3 days post-injury, and a return to control levels at 21 days post-injury in wild-type mice. In *EFmKL46* mice, however, *Klotho* gene expression was elevated over the entire regeneration period, and muscle fiber size significantly increased by 139% between 7 and 21 days post-injury, while wild-type mice showed only a 60% increase size during the same period of muscle repair (82). Taken together, these findings suggest that upregulation of locally expressed Klotho in skeletal muscle following injury is critical for effective muscle regeneration and this process is blunted in aged muscle. Several observations indicate that at least some of these effects of Klotho on skeletal muscle are mediated by Klotho antagonism of Wnt signaling.

Wnt signaling plays an essential role in muscle satellite cell differentiation and self-renewal (141). In the context of aging, increased canonical Wnt signaling results in a conversion of muscle satellite cells from a myogenic to a fibrogenic lineage, leading to muscle fibrosis rather than functional regeneration (142). In CKD, previous studies have shown increased muscle fibrosis in both mice models and humans, though a direct role of Wnt signaling has not been established (143–145). Klotho has previously been shown to interact with several Wnt ligands, and sKlotho is known to inhibit Wnt signaling (96–98). Furthermore, *in vitro* treatment with sKlotho in isolated muscle fibers was shown to inhibit Wnt signaling (33), and increased *Klotho* following muscle injury was accompanied by reduced Wnt target genes (82). Therefore, Klotho deficiency observed in aging and CKD may result in increased Wnt signaling, leading to fibrosis. However, other fibrotic pathways may also play a role. Circulating TGF-β1 is another pro-fibrotic factor increased during aging and CKD, resulting in fibrosis and impairing myogenesis (143, 146, 147). A study by Doi et al. (99) showed that sKlotho inhibits TGF-ß1 signaling by binding to its receptor, TGF-ßR2, preventing renal fibrosis. In *mdx* mice, expression of a *Klotho* transgene reduced the expression of Wnt target genes and TGF-ß1, which corresponded with reduced skeletal muscle fibrosis (140). Therefore, Klotho may protect skeletal muscle from fibrosis by antagonizing both Wnt and TGF-ß1 signaling.

It is well-known that aging is accompanied by a decrease in telomere length, which can impair tissue regeneration and is associated with many age-related diseases including CKD, cardiovascular disease, and diabetes, among others (55, 148, 149). Limited evidence suggests that telomere length in peripheral blood mononuclear cells is associated with sarcopenia (150, 151). However, the role of skeletal muscle telomere length in the development of sarcopenia remains unclear and the mechanisms that regulate telomere length in skeletal muscle tissue are unknown (152, 153). Evidence suggests that Klotho deficiency is highly associated with lower proliferation, lower telomerase activity, and shorter telomere length in adipose-derived stem cells (154). No study to date, however, has evaluated the relationship between Klotho levels and telomere length in skeletal muscle or whether skeletal muscle telomere length *in vivo* plays a role in the development of sarcopenia in aging and CKD.

### Klotho on Mitochondrial Function and Oxidative Stress

During “normal” aging, skeletal muscle mitochondria undergo various changes, such as abnormal enlargement and shape, shortened mitochondrial cristae and vacuolization of the matrix, decreased mitochondrial density, increased reactive oxygen species (ROS) production, decayed mitochondrial DNA, and increased mitochondria-mediated apoptosis (155). These age-related changes in mitochondrial function are thought to play a role in sarcopenia. Similarly, changes in skeletal muscle mitochondria have been implicated in the pathophysiology of CKD-associated muscle dysfunction (27, 156). Moreover, mitochondrial biogenesis is activated early during muscle regeneration and Klotho may play a key role during this process (34, 157).

Genetic knockdown of *Klotho* in young muscle progenitor cells was shown to result in pathogenic mitochondrial ultrastructure, decreased mitochondrial bioenergetics, mitochondrial DNA damage, and increased senescence (34). This suggests that Klotho is critical for the maintenance of muscle satellite cell mitochondrial ultrastructure, thereby limiting mitochondrial DNA damage and mitochondrial ROS production. However, the direct role of Klotho on muscle mitochondrial function is poorly understood. A recent study found that Klotho treatment in a CKD mouse model significantly preserved mitochondrial mass, inhibited mitochondrial ROS production, and restored the expression of mitochondrial respiration chain complex subunits in the kidneys (158). Moreover, Klotho significantly inhibited Wnt1- and Wnt9a-induced mitochondrial injury *in vitro*. These results suggest that Klotho's protective effects on mitochondria may be mediated by inhibiting Wnt signaling (158). Healthy mitochondria within muscle satellite cells are necessary for satellite cell activation and contribution to functional skeletal muscle regeneration (159). Therefore, Klotho deficiency may also impair muscle regeneration *via* decreased satellite cell mitochondrial integrity and function.

Elevated oxidative stress has been shown to contribute to sarcopenia in both aging (160) and CKD (161). Accordingly, Klotho deficiency has been shown to increase endogenous ROS generation and accentuate oxidative stress (162). To combat the harmful effects of ROS, mitochondria have an antioxidant defense system, which include manganese superoxide dismutase (MnSOD), catalase, and glutathione peroxidase (163). These enzymes scavenge ROS to maintain cellular homeostasis. Klotho may protect skeletal muscle fibers from oxidative stress by regulating forkhead box protein O (FoxO) transcription factors. FoxO transcription factors are downstream targets of the insulin/IGF-1 signaling pathway, the latter which is inhibited by Klotho (6, 101). As such, Klotho-mediated suppression of insulin/IGF-1 signaling activates FoxO proteins by inhibiting FoxO phosphorylation and promoting its nuclear translocation (101). Nuclear FoxO then directly binds to the MnSOD promoter and up-regulates its expression, thereby promoting antioxidant protection (100). Yamamoto et al. (101) found that Klotho deficient mice and *Klotho* overexpressing mice had muscle MnSOD protein levels of ~77 and ~234%, respectively, when compared to wild-type mice, suggesting that higher Klotho levels result in higher skeletal muscle MnSOD protein levels. Therefore, Klotho may further support musculoskeletal health by enhancing antioxidant protection in skeletal muscle.

### Klotho and Inflammation

In aging and CKD, chronic low-grade systemic inflammation accelerates muscle protein degradation and inhibits muscle protein synthesis, resulting in a negative net protein balance and muscle atrophy (26). In a state of chronic low-grade systemic inflammation, Tumor Necrosis Factor (TNF)-α inhibits myogenic differentiation and promotes protein degradation *via* induction of the Nuclear Factor (NF)κβ pathway (164). Activation of NFκβ signaling has been shown to induce significant muscle loss, as measured by increases in amino acid excretion and tyrosine turnover in isolated muscles (165). Inflammation-induced NFκβ signaling leads to muscle wasting through the activation of the ubiquitin–proteasome system (UPS). This system is thought to be the major cause of muscle wasting in aging (18) and CKD (26). Protein degradation by the UPS involves a series of enzymatic steps that link the cofactor, ubiquitin, onto proteins. Ubiquitinized proteins are then transferred to the proteasome complex to be degraded into short peptides and subsequently recycled as free intracellular amino acids (166). However, TNF-α plays a key role in muscle regeneration following acute injury (167). Thus, while chronic low-grade systemic inflammation contributes to muscle loss, local production of TNF-α by myocytes and tissue macrophages following muscle injury is necessary for muscle repair.

Previous investigations have shown that inflammation associated with CKD, diabetes, colitis or endotoxemia can significantly suppress Klotho expression in the kidneys (168–170) and myocardium (102). Studies show that TNF-α acts directly on kidney cells to reduce renal Klotho expression (169, 171). Since the kidneys are considered to be the primary source of the circulating protein, this would likely result in reduced sKlotho. Moreover, Wehling-Henricks and colleagues (172) showed that the significant reductions observed in Klotho mRNA in mouse *mdx* muscle coincide with significant increases in muscle expression of TNF-α and interferon (IFNγ), suggesting that proinflammatory cytokines could reduce Klotho expression in muscle. Klotho mRNA expression in quadriceps tissue of TNF-α-null mice was more than two-fold greater than wild-type muscle, and treatment with recombinant TNF-α and IFNγ reduced muscle Klotho mRNA by half *in vitro* (172). However, stimulation with either proinflammatory (TNF-α and IFNγ) or anti-inflammatory (IL-10 and IL-4) cytokines increases Klotho expression in macrophages *in vitro* (172). These findings suggest that while a pro-inflammatory environment in dystrophic muscle can reduce Klotho expression in muscle, it may increase macrophage-derived Klotho. The mechanisms through which macrophages in dystrophic muscle escape Klotho silencing is unclear.

A previous study showed that treatment with recombinant Klotho to endotoxemic mice reduced activation of NFκβ pathway and downregulated the concentration of IL-1ß, IL-6, and TNF-α in plasma and the myocardium (102). However, *mdx* mice overexpressing Klotho (*KL*+*/mdx)* exhibited higher TNF-α mRNA in skeletal muscle compared to wildtype and *mdx* mice, and stimulation with recombinant Klotho increases the production of TNF-α by macrophages *in vitro* (172). Moreover, the pro-proliferative effects of Klotho on myoblasts were mitigated by anti-TNF-α treatment. TNF-α generated from infiltrating macrophages and injured muscle fibers has been shown to enhance satellite cell proliferation and differentiation (167). Together, these findings suggest that while Klotho can mitigate chronic low-grade systemic inflammation, it may enhance muscle regeneration by promoting local TNF-α production during injury.

**Figure 1:**
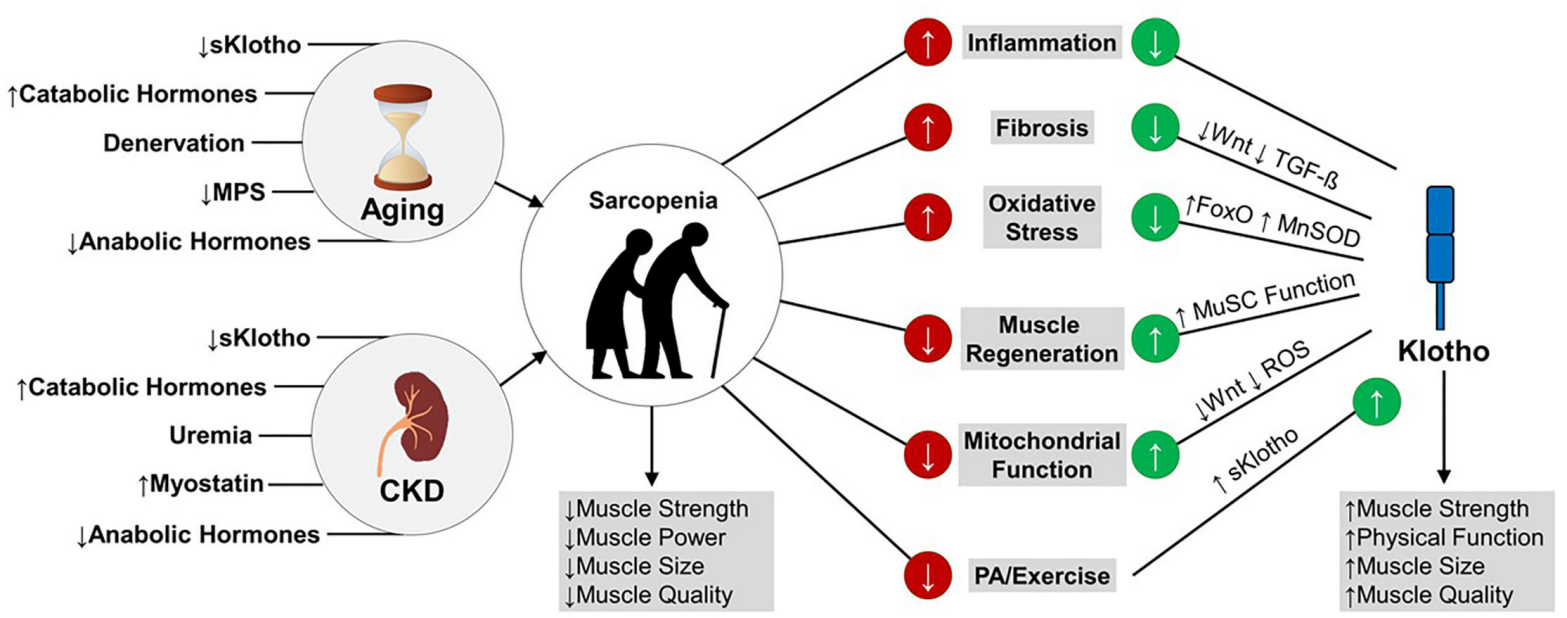
Mechanisms of Klotho's effects on muscle function in aging and CKD. Age- and CKD-related declines in skeletal muscle strength, power, size, and quality lead to sarcopenia. These declines in muscle structure and function are partly driven by increased inflammation and oxidative stress, impaired muscle regeneration in favor of fibrosis, and impaired mitochondrial function. Emerging data suggest that enhancing Klotho levels may reverse sarcopenia by downregulating pro-fibrotic pathways, enhancing muscle satellite cell and mitochondrial function, and downregulating oxidative stress and inflammation. Accordingly, higher circulating levels of Klotho are associated with greater muscle strength and physical function, higher lean mass, and higher muscle quality. Klotho levels have been shown to increase in response to exercise. MPS, muscle protein synthesis; TGF-ß, Transforming Growth Factor-ß; FoxO, Forkhead box protein O; MnSOD, Manganese Superoxide Dismutase; MuSC, Muscle Satellite Cell; ROS, Reactive Oxygen Species; sKlotho, soluble Klotho; PA, Physical Activity.

In summary, several pathophysiological mechanisms of sarcopenia are regulated by the extrarenal functions of Klotho, including muscle satellite cell function, mitochondrial function, oxidative stress, and inflammation. In a state of a Klotho deficiency, such as CKD and older age, enhancing circulating levels of Klotho and endogenous production of Klotho in skeletal muscle may prevent or reverse sarcopenia by preventing skeletal muscle fibrosis in favor of effective regeneration, improving muscle mitochondrial bioenergetics, and protecting muscle against oxidative damage and systemic inflammation (Figure 1). Strategies for enhancing Klotho levels are discussed in a later section.

## Klotho and Cardiovascular Functional Capacity

A characteristic feature of the aging process is a progressive and cumulative decline in the integrated metabolic machinery required to perform optimal aerobic work. CKD patients develop a phenotype that recapitulates many of the features of accelerated aging, involving impairment of multiple organ systems integral to physical function (54, 55). As CKD progresses, impairment of a network of integrated organs including the skeletal muscle as well as the bone (involving bone-mineral disease), heart (left ventricular hypertrophy and cardiac fibrosis), vasculature (arteriosclerosis and calcification), lungs (impaired lung function) and widespread molecular and ultrastructural changes collectively contribute to failure of the oxygen transport system (173). The interaction between these organ systems is well-illustrated by the Fick equation and describes the functional interdependence between the skeletal muscle system with the heart and lungs:


$$\begin{array}{l} \text{V}{\text{O}}_{2}=\text{HR}\times \text{SV}\times \left(\text{Ca}{\text{O}}_{2}-\text{Cv}{\text{O}}_{2}\right) \end{array}$$

where HR is heart rate, SV is the stroke volume, CaO_2_ is arterial oxygen content, and CvO_2_ is mixed venous oxygen content (174). These complex alterations can therefore be collectively assessed using state-of-the-art cardiopulmonary exercise testing (CPET) that enables objective quantitation of oxygen uptake (VO_2_) during incremental exercise testing. Assessment of VO_2_ at maximal or peak exercise (VO_2_max and VO_2_peak) has now been widely accepted as a robust index of cardiovascular functional capacity, and importantly considers the contribution resulting from impairment of the skeletal muscle system in disease states.

Healthy aging is associated with a progressive decline in VO_2_peak, which accelerates markedly with each successive decade (175). To-date, few studies if any have assessed the link between Klotho and VO_2_peak in health or CKD. This is worrying, particularly given the limitations of single surrogate markers for tracking or predicting musculoskeletal disease activity or impairment of physical function. Moreover, assessment of functional capacity by CPET under incremental exercise load is reflective of dynamic organ system interactions in health and the ability to respond to physiological and pathological stress (174). This has therefore significant advantages over conventional physical function assessments such as the 6 min walk tests (6MWT) for evaluating the role of Klotho in regulating exercise limitations. Given the pleiotropic role of Klotho and its apparent role as a central regulator of aging in diverse organ systems, a significant advantage of examining endpoints obtained by various modalities of CPET in the study of Klotho is that the technology permits assessment of the individual organ system limiting gas exchange. For example, invasive CPET testing involving CPET coupled with pulmonary artery and radial artery catheterization enables more detailed interrogation of the role of Klotho in regulating critical components of the Fick equation, including cardiopulmonary hemodynamic and peripheral O_2_ extraction analyses (176). This appears highly relevant, given Klotho's well-known role in regulating the development of arterial calcification (104, 170) and mitochondrial health (34, 158) for example. We postulate that deficiency of Klotho could therefore lead to declines in skeletal muscle oxygen uptake, however no studies have directly examined this to-date.

## Regulatory Role of Exercise on Klotho: is Klotho a Myokine?

According to the American College of Sports Medicine (ACSM) Position Stand on Exercise and Physical Activity for Older Adults, the physiological benefits of physical activity and exercise include increases in cardiorespiratory fitness, improved bone health, and increased muscle strength and power (177). A review by Avin and colleagues (81) highlighted the parallels between the age-related processes regulated by Klotho and those regulated by exercise, driving the hypothesis that skeletal muscle contractile activity may regulate Klotho expression and that this exercise-related increase in Klotho is partly responsible for the health benefits of exercise. Skeletal muscle fibers are known to produce more than 3,000 secreted factors, including proteins, growth factors, and cytokines capable of exerting autocrine, paracrine, or endocrine effects. These muscle-derived molecules are termed “myokines” and their production can increase during muscle contractions, myogenesis and muscle remodeling, or in response to exercise training (178). Though Klotho is currently not formally known as a myokine, emerging evidence suggests that Klotho may fit the description.

Limited evidence suggests that sKlotho is increased following acute aerobic exercise in both mice and humans (81, 130, 179–182). However, these studies are limited by relatively small sample sizes and/or poor study design. Less is known regarding the impact of acute exercise on skeletal muscle expression of Klotho. One study found that both Klotho mRNA and protein expression in skeletal muscle were blunted immediately following exhaustive exercise in mice and then increased 3 to 5 days post-exercise (183). It should be noted however, that Klotho protein expression was only detected at 65 kDa and 95 kDa in skeletal muscle, the latter of which is inconsistent with previous literature (140). Given this limited evidence, it is currently unclear whether acute exercise promotes endogenous Klotho production in skeletal muscle. It is also unknown whether skeletal muscle is a source of sKlotho following exercise. Studies evaluating the effects of acute exercise on Klotho levels are summarized in Table 2.

**Table 2:** Effects of acute exercise on Klotho expression.

**Reference**	**Study design**	**Population**	**Sample and technique**	**Key findings**
Avin et al. (81)	-45-min of treadmill running at ~70% VO_2_max. -Klotho measured pre- and post-exercise	Young (3–4 mo.) and aged (22–24 mo.) C57B16/J mice	-Plasma -ELISA (IBL Ltd., Takasaki, Japan)	↑ in sKlotho immediately post-exercise with a smaller increase observed in aged mice.
Avin et al. (81)	-Young group: 1 hour of treadmill walking at 55% VO_2_max -Older group: 1 hour of cycling at 45% VO_2_max -Klotho measured pre- and post-exercise at baseline and after 16 and 12 weeks of exercise training in the young and older group, respectively.	Young (age 36.0 ± 7.0 years; *n* = 12) and older (age 68.3 ± 3.0 years; *n* = 7) sedentary women.	-Serum -ELISA (IBL Ltd., Takasaki, Japan)	-No significant changes in sKlotho following acute exercise at baseline. -↑ in sKlotho following acute exercise after exercise training in the younger group. -No significant changes in sKlotho following acute exercise after exercise training in the older group.
Iturriaga et al. (184)	-Cardiorespiratory exercise group (CR): 30 min of treadmill running at 75% VO_2_max. -Strength exercise group (ST): 5 × 20 weighted depth jumps with 2 min rest intervals between sets and 10s of rest between each jump. -Klotho measured pre- and post-exercise in both groups and at 24h, 48h, and 72h post-exercise in ST only.	46 physically active men in the CR group (age 35.8 ± 8.1 years) and 45 physically active men in the ST group (age 23.3 ± 3.9 years).	-Plasma -ELISA (IBL Ltd., Japan)	-↑ in sKlotho immediately post-exercise in CR compared to pre-exercise and compared to ST. -↓ in sKlotho immediately post-exercise in ST, followed by an ↑ at 24 h and 48 post-exercise compared to pre-exercise.
Rahimi et al. (182)	Standard Bruce protocol on a treadmill.	Healthy non-athlete women (*n* = 10, age 32 ± 7 years) and healthy female athletes (*n* = 10, age 31 ± 9 years).	-Plasma -ELISA (IBL, D-22335, Hamburg, Germany)	-Athletes have a higher level of sKlotho than non-athletes at baseline and post-exercise. -↑ in sKlotho immediately after exercise in both athletes and non-athletes compared to baseline.
Ramez et al. (185)	−5 consecutive days of training -HIIT: 6 × 2 min at 85–90% VO_2_max and 5 × 2 min at 50–60%VO_2_max. -MICT: running an identical distance as the HIIT group at 70% VO_2_max. -Control: sat on the treadmill belt. -IR: ligation of the LAD for 30 min followed by 24 h reperfusion. -Sham: same surgical procedure without LAD ligation. -Klotho measured 24 h after the last training session.	-Male Wistar rats (wt. 250–300g), ages 8–10 wks. -Randomized into 7 groups: control (*n* = 8), HIIT (*n* = 8), MICT (*n* = 8), Sham (*n* = 14), IR (*n* = 14), HIIT+IR (*n* = 14), and MICT+IR (*n* = 14).	-Plasma -ELISA (Bioassay Technology Laboratory, Shanghai crystal day biotech Co, LTD; Shanghai)	-↑ in sKlotho following both exercise training protocols compared to control but was significantly higher in HIIT than MICT -Animals in the sedentary IR group had significantly lower levels of sKlotho when compared to animals with an IR who performed exercise.
Ramez et al. (186)	−5 consecutive days of training -HIIT: 6 × 2 min at 85–90% VO_2_max and 5 × 2 min at 50–60%VO_2_max. -IR: ligation of the LAD for 30 min followed by 24 h reperfusion. -Sham: same surgical procedure without LAD ligation. -Klotho measured 24 h after the last training session.	-Male Wistar rats (wt. 250–300 g), ages 8–10 wks. -Randomized into 5 groups: control, HIIT, Sham, IR, and HIIT+IR.	-Plasma and myocardial tissue -ELISA (Bioassay Technology Laboratory, Shanghai crystal day biotech Co, LTD; Shanghai). -Western blot (polyclonal antibody, Thermo Fisher Scientific, Waltham, MA, USA)	-↑ in sKlotho following HIIT compared to all groups. -↑ in myocardial levels of 130 kDa Klotho after HIIT compared to the control group. -myocardial levels of 130 kDa Klotho were higher in the HIIT+IR group compared to the sedentary IR group.
Rao et al. (183)	-Treadmill running: 10m/min for 15 min, then 15 m/min for 15 min, then 20 m/min for 15 min, then 24 m/min until exhaustion (defined as, hindlimbs touching electrical grid for more than 10 s)	-Male C57BL6 mice, ages 8 wks. -Divided into 6 groups: sedentary (*n* = 6), immediately post-(*n* = 7), 12 h post-(*n* = 7), 24 h post-(*n* = 7), 3 days post-(*n* = 7), and 5 days post-(*n* = 6) exercise.	-Gastroc., tib. anterior, quads, epididymal WAT, inguinal WAT, kidneys, brain, lungs, and liver. -qPCR (forward primer: 50- CACGCCGAGCAAGACTCACTG-30; reverse primer: 50-TTGATGTCGTCCAACACGTAGGC-30) -Western Blot (AF1819, R&D, MN, United States)	-Kidneys: Klotho mRNA ↓ at 12 h post-exercise and ↑ 3- and 5-days post-exercise compared to sedentary. 65 kDa and 130 kDa Klotho expression ↓ immediately post-exercise compared to sedentary and ↑ 5 days post-exercise compared to other timepoints but not compared to sedentary. -Muscle: ↓ Klotho mRNA in quads and gastroc. immediately and 12 h post-exercise, and ↑ 3- and 5-days post-exercise compared to sedentary. In the tib. anterior, Klotho mRNA ↓ post-exercise compared to sedentary. 130 kDa Klotho was not found in muscle. No significant changes in 65 kDa Klotho expression in muscle.
				↑ in 95 kDa Klotho expression in muscle following exercise compared to sedentary.
Tan et al. (180)	Standard Bruce protocol on a treadmill.	−10 healthy adults (men *n* = 5) -median (IQR) age = 47.5 (44–51) years.	-Serum -ELISA (IBL Ltd., Gunma, Japan)	- ↑ in sKlotho immediately post-exercise and return to baseline at 30 min post-exercise.

*VO_2_max, maximal oxygen uptake; ELISA, enzyme-linked immunosorbent assay; sKlotho, soluble Klotho; IBL, Immuno-Biological Laboratories; HIIT, high-intensity interval training; MICT, moderate-intensity continuous training; IR, ischemia-reperfusion; LAD, left anterior descending coronary artery*.

Several studies have reported a positive association between aerobic fitness and sKlotho (122, 182, 187–189). Accordingly, aerobic exercise training interventions have been shown to increase sKlotho and tissue expression of Klotho. Significant increases in sKlotho have been reported following 12 weeks of aerobic exercise in trained young women (182), postmenopausal women (129) and in patients with coronary artery disease (190). Studies have reported increased sKlotho as well as increased mRNA and protein expression of Klotho in the kidney and brain following aerobic exercise training in Sprague Dawley rats (191, 192). Another study found that five consecutive days of high-intensity interval exercise increased sKlotho and myocardial expression of Klotho protein in Wistar rats (186). Interestingly, a study comparing master runners (40–65 y) from endurance and sprint events to untrained young and age-matched controls found that the sprint athletes had significantly higher sKlotho compared to the endurance athletes and untrained age-matched controls (133). This suggests that strength/power training may be superior to endurance training for maintaining sKlotho levels, and that training variables (i.e., type, intensity, and duration) may influence exercise training-induced changes in Klotho expression.

Studies evaluating the influence of exercise training variables have yielded mixed results. A 12-week randomized controlled trial assigned sedentary middle-aged adults to 4 different groups: a control group (no exercise), a group that performed combined aerobic and resistance training following the physical activity recommendations from the World Health Organization (PAR), a high-intensity interval training group (HIIT), and a high-intensity interval training group adding whole-body electromyostimulation (HIIT-EMS) (193). All three exercise interventions resulted in a significant increase in sKlotho from baseline (PAR: 714.3 ± 294.5 to 1,055.4 ± 435.9 pg/mL; HIIT: 788.5 ± 276.8 to 1,057.1 ± 273.3 pg/mL; HIIT-EMS: 808.5 ± 499.0 to 1,259.7 ± 613.1 pg/mL; all *p* < 0.001), while no differences were found in the control group (922.5 ± 290.3 to 862.9 ± 364.7 pg/mL; *p* = 0.142) or between exercise interventions. Ramez et al. (185) showed that high-intensity interval exercise led to significantly greater increases in sKlotho compared to moderate intensity continuous exercise in Wistar rats. Six months of aerobic exercise performed at 65–70% of VO_2_max significantly increased VO_2_max and sKlotho in young untrained men, while the same intervention performed at 45–50% VO_2_max did not increase either, suggesting that there may be an intensity threshold for increasing sKlotho in response to training (188). Conversely, Middelbeek et al. (194) recently found that 2 weeks of moderate intensity training (MIT) led to significant increases in sKlotho (Pre: 3.8, 95% CI 2.5–5.0 ng/mL; Post: 5.9, 95% CI 4.6–7.2 ng/mL) in sedentary middle-aged men while sprint interval training (SIT) did not (Pre: 4.9, 95% CI 3.8–6.1 ng/mL; Post: 4.3, 95% CI 3.2–5.4 ng/mL), despite both interventions resulting in similar improvements in VO_2_peak. Therefore, the influence of training variables on exercise-induced changes in sKlotho remains unclear.

Only two studies have evaluated the effects of exercise training on sKlotho on individuals with CKD and both were performed on hemodialysis patients. One study evaluated the effects of 16 weeks of intradialytic exercise consisting of combined aerobic and resistance exercise performed 3 days per week compared to inactive controls (195). The exercise group showed a significant increase in sKlotho (Pre: 360.59 ± 107.55 pg/mL; Post: 397.25 ± 133.36 pg/mL) while the inactive group showed a significant decrease after the intervention (Pre: 392.75 ± 143.22 pg/mL; Post: 362.93 ± 147.05 pg/mL). Neves and colleagues (196) found that 6 months of resistance training performed 1 hour before dialysis 3 days per week significantly elevated sKlotho levels compared to baseline (Pre: 148 ± 70 pg/mL; Post: 279 ± 51 pg/mL) and to the control (Pre: 136 ± 84 pg/mL; Post: 108 ± 63 pg/mL). Moreover, the authors also reported enhanced handgrip strength and bone mineral density following resistance training. Taken together, current evidence suggests that both aerobic and resistance exercise can promote significant increases in circulating Klotho along with improvements in physical function. Therefore, exercise may be an effective strategy to prevent and/or reverse Klotho deficiency. Moreover, exercise-induced increases in circulating Klotho may underlie some of the beneficial effects of exercise on health and physical function. Studies evaluating the effects of exercise training on Klotho levels are summarized in Table 3.

**Table 3:** Effects of exercise training on Klotho expression.

**Reference**	**Study design**	**Population**	**Sample and technique**	**Key findings**
Amaro-Gahete et al. (193)	−12 weeks -control: no exercise -PA recommendations from WHO: 3 days/wk of both aerobic (150 min/wk at 60–65% HRR) and resistance exercise (~60 min/wk of 40–50% 1RM) -HIIT: 2 days/wk with two different protocols: 40–65 min/wk at >95% VO_2_max using a treadmill (session A) and >120% VO_2_max using a circuit workout (session B) -HIIT-EMS: same as HIIT plus whole-body electromyostimulation	Sedentary middle-aged adults (*n* = 68, 32 men, age 53.4 ± 5.0) randomly assigned to control (*n* = 15), PAR (*n* = 17), HIIT (*n* = 17), and HIIT-EMS (*n* = 19)	-Plasma -ELISA (Demeditec, Kiel, Germany)	-↑ in sKlotho following all exercise interventions compared to baseline and control. -No significant difference between the training groups.
Dalise et al. (192)	-4 weeks, 5 days per week -Treadmill running at 80% of their maximal baseline activity or NMES at three different doses: -Low: 15 min running or 1 set of 10 stimulations -Medium duration: 30 min running or 2 sets of 10 stimulations -high duration: 60 min running or 3 sets of 10 stimulations -Control: no treatment	Male Sprague-Dawley Rats, 12 weeks of age randomized into 7 treatment groups: low (*n* = 5), medium (*n* = 5), and high (*n* = 5) running, low (*n* = 5), medium (*n* = 5), and high (*n* = 5) NMES, and control (*n* = 10).	-Hippocampal tissue -ELISA (CSB-E14958r, CUSABIO) -qPCR (#Rn00580123_m1, Applied Biosystems	-↑ in sKlotho levels for all running conditions compared to control, with the highest level being produced by the medium condition. -NMES did not significantly affect sKlotho levels. -↑ in Klotho mRNA expression in the hippocampus in all exercise groups compared to control except the low running group.
Fakhrpour et al. (195)	-16 weeks, 3 days per week -Combined intradialytic aerobic and resistance training -Aerobic exercise: progressive cycling beginning at 10 min duration at a comfortable pace with 5–10 min increases per session with a goal of 45 min per session at 12–14 RPE. -Resistance training: lower extremity exercises at an intensity of 9–15 RPE starting at ~20% 1RM for 2 sets of 12 reps and increased to 3 sets as tolerable. Resistance increased to 40, 55, and 65% 1RM as tolerated.	Hemodialysis patients (*n* = 45, 38 men, age 61.0 ± 9.0) randomly assigned to control (*n* = 21) and intradialytic exercise training (*n* = 24).	-Serum -ELISA (IBL Ltd., Tokyo, Japan)	-↑ in sKlotho compared to baseline and compared to control. -↓ in sKlotho in the control patients.
Gaitan et al. (187)	-26 weeks, 3 days per week -Usual Physical Activity (UPA): maintained their usual level of PA -Enhanced Physical Activity (EPA): Participated in supervised, progressive, moderate-to-vigorous intensity aerobic exercise.	Sedentary middle-aged adults with family history of Alzheimer's disease (*n* = 23) were randomized to either UPA (*n* = 12, 6 men, age 63.9 ± 5.2) or EPA (*n* = 11, 6 men, age 65.9 ± 4.0 years).	-Serum -ELISA (IBL Ltd., Takasaki, Japan)	-No changes in sKlotho in either group. -Changes in sKlotho were positively correlated with changes in VO_2_peak.
Ji et al. (191)	-48 weeks, 5 days per week, 60 min per day -Intermittent aerobic exercise (IAE): 10 m/min (40–50% VO_2_max) for 10 min, 25 m/min (80–90% VO_2_max) for 7 min and 15 m/min (50–60% VO_2_max for 3 min, repeat 2x. -Continuous aerobic exercise (CAE): 16 m/min (50–60% VO_2_max). -Control: no exercise	Male Sprague-Dawley Rats, 3 months old randomized to control (*n* = 30), IAE (*n* = 31), and CAE (*n* = 32)	- Kidneys and brain. -RT-qPCR (upstream primer: 5'-ATC CGG CCT CAG ATA ACC TT-3'; downstream primer: 5'-CCA CCA CTG GAG TGA TGT TG-3') -Western blot (ab203576; 1:2,000, Abcam, Cambridge, UK)	-↑ in Klotho mRNA and protein (116 kDa) expression in brain and kidneys in CAE and IAE compared to control. -No difference between exercise groups.
Matsubara et al. (129)	-12 weeks, 3–4 days per week (2–3 supervised sessions and home-based training) -Cycling and walking for 30 min/day at 60% max HR -Increased to 40–60 min/day at 70–80% max HR as tolerated -Control: no exercise	Healthy and postmenopausal women (*n* = 19, aged 62 ± 2.5) 50–76 years old) divided into control (*n* = 8) and exercise group (*n* = 11).	-Plasma -ELISA (IBL Ltd., Tokyo, Japan)	-↑ in sKlotho in the exercise group compared to baseline. -No change in sKlotho in the control group.
Middelbeek et al. (194)	−2 weeks, 6 total training sessions -Sprint interval training (SIT): 6 × 30 s maximal cycle ergometer sprints with 4 min of recovery -Moderate intensity continuous training (MIT): Cycling at 60% VO_2_peak for 40 min (sessions 1–3) and 60 min (sessions 3–6).	Healthy middle-aged men (*n* = 22, age 48 ± 5 years) randomized into SIT (*n* = 12) and MIT (*n* = 10)	-Serum -ELISA (NeoBioLab, #HK0034)	-↑ in sKlotho following MIT program -No change in sKlotho following SIT program
Neves et al. (196)	-6 months, 3 days per week, ~40 min per day, ~1 hour before dialysis -Dynamic resistance training (DRT): Full body resistance training using elastic bands and free weights. -Isometric resistance training (IRT): same exercise as DRT but performed isometrically.	Maintenance hemodialysis patients were randomized into control (*n* = 60, 30 men, age 55 ± 12 years), DRT (*n* = 66, 33 men, age = 58 ± 15 years), and IRT (*n* = 67, 31 men, age = 56 ± 19)	-Plasma -ELISA (IBL Ltd., Japan)	-↑ in sKlotho following DRT compared to baseline, IRT and control. -↑ in sKlotho following IRT compared to control.
Rahimi et al. (182)	-12 weeks, 3 days per week -Water Aerobic program: Starting at 30 min (60% of max HR) then progressing to 40–60 min (70–80% max HR).	Healthy non-athlete women (*n* = 10, age 32 ± 7 years) and healthy female athletes (*n* = 10, age 31 ± 9 years).	-Plasma -ELISA (IBL, D-22335, Hamburg, Germany)	-↑ in sKlotho 24 hours after training intervention in athlete group compared to baseline and non-athletes. -No change in sKlotho following training intervention in non-athlete group.
Saghiv et al. (189)	-12 months, 4–5 times per week -supervised aerobic programs at 60–75% work capacity.	CAD patients that participated in the exercise program (*n* = 60, age 53.0 ± 2.0 years), untrained CAD patients (*n* = 60, age 52.6 ± 2.0 years), and untrained healthy men (*n* = 40, age 53.6 ± 1.5 years)	-Serum -ELISA (IBL Ltd., Japan)	-↑ sKlotho was observed the trained CAD patients compared to both untrained CAD patients and untrained healthy men.
Saghiv et al. (190)	-12 weeks, 4–5 days per week, 45 min per day. -Individualized cardiac rehabilitation programs at 75–80% of max HR. -Control: no exercise intervention	CAD patients (*n* = 41, 30 men, age 59.6 ± 2.2 years) who performed cardiac rehabilitation and age-matched control CAD patients (*n* = 17, 12 men, age 61 ± 2.4 years).	-Serum -ELISA (IBL Ltd., Japan)	-↑ in sKlotho levels following the exercise program compared to baseline. -No change in sKlotho in the control group.
Saghiv et al. (188)	-6 months, 4 days per week, 45 min per day. -Treadmill running -Low training intensity (LTI): 40–50% VO_2_max High training intensity (HTI): 65–70% VO_2_max	Young, healthy, untrained men (*n* = 60, age 27.0 ± 1.1 years) were randomized into LTI (*n* = 30) and HTI (*n* = 30).	-Serum -ELISA (IBL Ltd., Japan)	-↑ in sKlotho in the HTI group at 2 and 4 months of training compared to baseline. -sKlotho was higher in HTI compared to LTI at 2, 4, and 6 months of training. -no change in sKlotho was observed for the LTI group.

*PA, physical activity; WHO, World Health Organization; HRR, heart rate reserve; ELISA, enzyme-linked immunosorbent assay; IBL, Immuno-Biological Laboratories; sKlotho, soluble Klotho; NMES, neuromuscular electrical stimulation; RPE, ratings of perceived exertion; VO_2_peak, peak oxygen uptake; VO_2_max, maximum oxygen uptake; CAD, coronary artery disease*.

## Klotho Supplementation and Physical Function

Counteracting the decrease in Klotho that occurs with increasing age and CKD represents a promising strategy to prevent and reverse sarcopenia and cardiovascular disease in these populations. Therapeutic approaches to enhance Klotho expression include the administration of recombinant Klotho protein, gene therapy, and agents that can increase endogenous Klotho production. Potential agents that can increase endogenous Klotho production include those that target Klotho synthesis, such as 1,25(OH)_2_D_3_, vitamin D receptor agonists, phosphate binders, angiotensin II receptor antagonists, peroxisome proliferator-activated receptor-γ agonists, androgens, and statins, and novel small molecules (121, 197–199). As mentioned earlier, exercise and physical activity may also enhance Klotho production, though the mechanisms behind exercise-induced increases in sKlotho have been largely unexplored. Several preclinical studies have evaluated Klotho-based therapies for the treatment of renal pathologies and have reported reduced renal damage, amelioration of renal fibrosis, reduced hypertension, and improved recovery from acute kidney injury (98, 200, 201). Additionally, Klotho therapy has also been shown to improve endothelial function (202), reduce vascular calcification (198), and ameliorate uremic cardiomyopathy in CKD models (203). Therefore, Klotho replacement therapy can target multiple organ systems that are integral to physical function, including the heart, vasculature, and potentially skeletal muscle.

A recent study by Clemens and colleagues (5) found that Klotho treatment *via* adeno-associated virus (AAV) in old (21–24 mo.) mice significantly reduced skeletal muscle fibrosis, reduced intramuscular lipid accumulation, enhanced muscle mitochondrial structure, and improved muscle strength and endurance. However, Klotho treatment did not improve muscle function in the oldest (27–29 months old) mice, suggesting that Klotho therapy may be effective in slowing the progression of sarcopenia early on but may not reverse sarcopenia in advanced age. Sahu et al. (34) demonstrated that systemic administration of recombinant Klotho significantly enhanced skeletal muscle force recovery and increased local expression of Klotho protein within the injured muscle of aged mice. Interestingly, the timing of administration was shown to be crucial. Daily intraperitoneal injections of recombinant Klotho on days 3–5 post-injury (the timeframe that corresponds to upregulation of Klotho in young mice) yielded the most improvement. Conversely, administration of Klotho from days 1–6 post-injury severely impaired functional recovery when compared to saline-injected controls (204). This finding suggests that more research is needed to determine the mechanisms of Klotho in enhancing muscle function.

## Conclusions and Future Directions

Despite several noteworthy advances in our understanding of Klotho, a number of critical gaps in our knowledge of its role and mechanisms in skeletal muscle remain. For instance, the distinct functional roles of the sKlotho isoforms in skeletal muscle and their molecular mechanisms are unknown. Moreover, the identity of the sKlotho receptor for FGF23-independent cellular effects in skeletal muscle is unclear. Limited evidence suggests that the gangliosides GM1 (monosialotetrahexosylganglioside) and GM3 (monosialodihexosylganglioside) present in lipid rafts may serve as membrane receptors for sKlotho (205). Since membrane ganglioside content has been previously linked to skeletal muscle regeneration (206), it is plausible that Klotho's effects on skeletal muscle are at least partially mediated by membrane gangliosides. Little is known regarding the functions of endogenous Klotho production in skeletal muscle tissue or whether autocrine or paracrine actions are involved in regulating muscle function. Also, the precise concentrations of sKlotho that are necessary to maintain or improve physical function are unknown and this is likely dependent on genetic variability between populations. Further research is needed to determine the role of Klotho levels on muscle function across the various stages of CKD and other disease populations. Elucidating the mechanisms of Klotho signaling in skeletal muscle may provide additional opportunities for treatment developments that can address the growing burden of sarcopenia and its progression to physical disability.

In summary, emerging evidence suggests that Klotho may be a key regulator of skeletal muscle function and may underlie some of the health benefits related to exercise. Regardless of etiology, declines in Klotho levels are associated with poor indices of musculoskeletal health and physical function. Limited data suggests that exercise may enhance Klotho production and higher fitness levels are associated with greater Klotho levels. Moreover, research in animal models suggests that Klotho-based therapy may enhance muscle function and reverse sarcopenia. However, several significant gaps in our knowledge of Klotho must first be overcome before we can harness its potential ergogenic benefits. An interdisciplinary collaborative research effort between Nephrologists, Exercise Physiologists and basic scientists is critically needed to determine the precise role of Klotho in regulating skeletal muscle function, elucidate the mechanisms of Klotho's functions in skeletal muscle, and explore the mechanistic link between exercise and Klotho production.

## Author Contributions

EA wrote the manuscript. AT generated the tables. KL, RM, KA, and AC provided the intellectual guidance and edited the manuscript. All authors contributed to the article and approved the submitted version.

## Funding

KL is a recipient of an NIH K23 (DK115683-03) Grant.

## Conflict of Interest

KL is Co-Founder of OVIBIO Corporation in Cambridge, MA. The remaining authors declare that the research was conducted in the absence of any commercial or financial relationships that could be construed as a potential conflict of interest.

## Publisher's Note

All claims expressed in this article are solely those of the authors and do not necessarily represent those of their affiliated organizations, or those of the publisher, the editors and the reviewers. Any product that may be evaluated in this article, or claim that may be made by its manufacturer, is not guaranteed or endorsed by the publisher.

## References

[1] Kuro-o M Matsumura Y Aizawa H Kawaguchi H Suga T Utsugi T. Mutation of the mouse klotho gene leads to a syndrome resembling ageing. Nature. (1997) 390:45–51. 10.1038/36285 9363890

[2] Nakatani T Sarraj B Ohnishi M Densmore MJ Taguchi T Goetz R. In vivo genetic evidence for klotho-dependent, fibroblast growth factor 23 (Fgf23)-mediated regulation of systemic phosphate homeostasis. FASEB J. (2009) 23:433–41. 10.1096/fj.08-114397 18835926 PMC2630784

[3] Phelps M Pettan-Brewer C Ladiges W Yablonka-Reuveni Z. Decline in muscle strength and running endurance in klotho deficient C57BL/6 mice. Biogerontology. (2013) 14:729–39. 10.1007/s10522-013-9447-2 24030242 PMC3851892

[4] Masuda H Chikuda H Suga T Kawaguchi H Kuro-o M. Regulation of multiple ageing-like phenotypes by inducible klotho gene expression in klotho mutant mice. Mech Ageing Dev. (2005) 126:1274–83. 10.1016/j.mad.2005.07.007 16144705

[5] Clemens Z Sivakumar S Pius A Sahu A Shinde S Mamiya H. The biphasic and age-dependent impact of klotho on hallmarks of aging and skeletal muscle function. Elife. (2021) 10:e61138. 10.7554/eLife.61138 33876724 PMC8118657

[6] Kurosu H Yamamoto M Clark JD Pastor JV Nandi A Gurnani P. Suppression of aging in mice by the hormone Klotho. Science. (2005) 309:1829–33. 10.1126/science.1112766 16123266 PMC2536606

[7] Crasto CL Semba RD Sun K Cappola AR Bandinelli S Ferrucci L. Relationship of low-circulating “anti-aging” klotho hormone with disability in activities of daily living among older community-dwelling adults. Rejuvenation Res. (2012) 15:295–301. 10.1089/rej.2011.1268 22530731 PMC3388499

[8] Semba RD Cappola AR Sun K Bandinelli S Dalal M Crasto C. Relationship of low plasma klotho with poor grip strength in older community-dwelling adults: the InCHIANTI study. Euro J Appl Physiol. (2012) 112:1215–20. 10.1007/s00421-011-2072-3 21769735 PMC3435096

[9] Semba RD Ferrucci L Sun K Simonsick E Turner R Miljkovic I. Low plasma klotho concentrations and decline of knee strength in older adults. J Gerontol Series A: Biomed Sci Med Sci. (2016) 71:103–8. 10.1093/gerona/glv077 26359247 PMC4706099

[10] Shardell M Semba RD Kalyani RR Bandinelli S Prather AA Chia CW. Plasma klotho and frailty in older adults: findings from the InCHIANTI Study. J Gerontol Series A. (2017) 74:1052–7. 10.1093/gerona/glx202 29053774 PMC6580690

[11] Shardell M Semba RD Kalyani RR Hicks GE Bandinelli S Ferrucci L. Serum 25-hydroxyvitamin D, plasma klotho, and lower-extremity physical performance among older adults: findings from the InCHIANTI study. J Gerontol Series A: Biomed Sci Med Sci. (2015) 70:1156–62. 10.1093/gerona/glv017 25748032 PMC4553717

[12] Koufaki P Mercer T. Assessment and monitoring of physical function for people with CKD. Adv Chron Kid Dis. (2009) 16:410–9. 10.1053/j.ackd.2009.08.010 19801131

[13] Painter P Roshanravan B. The association of physical activity and physical function with clinical outcomes in adults with chronic kidney disease. Curr Opin Nephrol Hypertens. (2013) 22:615–23. 10.1097/MNH.0b013e328365b43a 24100215

[14] Myers J Prakash M Froelicher V Do D Partington S Atwood JE. Exercise capacity and mortality among men referred for exercise testing. New Eng J Med. (2002) 346:793–801. 10.1056/NEJMoa011858 11893790

[15] Cawthon PM Fox KM Gandra SR Delmonico MJ Chiou C-F Anthony MS. Do muscle mass, muscle density, strength, and physical function similarly influence risk of hospitalization in older adults? J Am Geriatric Soc. (2009) 57:1411–9. 10.1111/j.1532-5415.2009.02366.x 19682143 PMC3269169

[16] Fusco O Ferrini A Santoro M Lo Monaco MR Gambassi G Cesari M. Physical function and perceived quality of life in older persons. Aging Clinic Experim Res. (2012) 24:68–73. 10.1007/BF03325356 22643307

[17] Khavjou OA Anderson WL Honeycutt AA Bates LG Razzaghi H Hollis ND. National health care expenditures associated with disability. Medical Care. (2020) 58. 10.1097/MLR.0000000000001371 32826747 PMC7505687

[18] Lang T Streeper T Cawthon P Baldwin K Taaffe DR Harris T. Sarcopenia: etiology, clinical consequences, intervention, and assessment. Osteoporosis Int. (2010) 21:543–9. 10.1007/s00198-009-1059-y 19779761 PMC2832869

[19] von Haehling S Morley JE Anker SD. An overview of sarcopenia: facts and numbers on prevalence and clinical impact. J Cachexia Sarcopenia Muscle. (2010) 1:129–33. 10.1007/s13539-010-0014-2 21475695 PMC3060646

[20] Delmonico MJ Harris TB Visser M Park SW Conroy MB Velasquez-Mieyer P. Longitudinal study of muscle strength, quality, and adipose tissue infiltration. Am J Clinic Nutr. (2009) 90:1579–85. 10.3945/ajcn.2009.28047 19864405 PMC2777469

[21] Goodpaster BH Park SW Harris TB Kritchevsky SB Nevitt M Schwartz AV. The loss of skeletal muscle strength, mass, and quality in older adults: the health, aging and body composition study. J Gerontol Series A: Biol Sci Med Sci. (2006) 61:1059–64. 10.1093/gerona/61.10.1059 17077199

[22] Reid KF Fielding RA. Skeletal muscle power: a critical determinant of physical functioning in older adults. Exerc Sport Sci Rev. (2012) 40:4–12. 10.1097/JES.0b013e31823b5f13 22016147 PMC3245773

[23] Wilkinson TJ Miksza J Yates T Lightfoot CJ Baker LA Watson EL. Association of sarcopenia with mortality and end-stage renal disease in those with chronic kidney disease: a UK Biobank study. J Cachexia, Sarcopenia Muscle. (2021) 12:586–98. 10.1002/jcsm.12705 33949807 PMC8200422

[24] Shafiee G Keshtkar A Soltani A Ahadi Z Larijani B Heshmat R. Prevalence of sarcopenia in the world: a systematic review and meta-analysis of general population studies. J Diabet Metabol Disord. (2017) 16:21. 10.1186/s40200-017-0302-x 28523252 PMC5434551

[25] Moorthi RN Avin KG. Clinical relevance of sarcopenia in chronic kidney disease. Curr Opin Nephrol Hypertens. (2017) 26:219–8. 10.1097/MNH.0000000000000318 28198733 PMC5860815

[26] Fahal IH. Uraemic sarcopenia: aetiology and implications. Nephrol Dial Transplant. (2013) 29:1655–65. 10.1093/ndt/gft070 23625972

[27] Gamboa JL Roshanravan B Towse T Keller CA Falck AM Yu C. Skeletal muscle mitochondrial dysfunction is present in patients with ckd before initiation of maintenance hemodialysis. Clin J Am Soc Nephrol. (2020) 15:926–36. 10.2215/CJN.10320819 32591419 PMC7341789

[28] Zhou Y Hellberg M Svensson P Höglund P Clyne N. Sarcopenia and relationships between muscle mass, measured glomerular filtration rate and physical function in patients with chronic kidney disease stages 3–5. Nephrol Dial Transplant. (2017) 33:342–8. 10.1093/ndt/gfw466 28340152

[29] Fitzgerald MD Tanaka H Tran ZV Seals DR. Age-related declines in maximal aerobic capacity in regularly exercising vs. sedentary women: a meta-analysis. J Appl Physiol. (1997) 83:160–5. 10.1152/jappl.1997.83.1.160 9216959

[30] Wilson TM Tanaka H. Meta-analysis of the age-associated decline in maximal aerobic capacity in men: relation to training status. Am J Physiol Heart Circulat Physiol. (2000) 278:H829–H34. 10.1152/ajpheart.2000.278.3.H829 10710351

[31] Howden EJ Weston K Leano R Sharman JE Marwick TH Isbel NM. Cardiorespiratory fitness and cardiovascular burden in chronic kidney disease. J Sci Med Sport. (2015) 18:492–7. 10.1016/j.jsams.2014.07.005 25127529

[32] Kreider RB Wilborn CD Taylor L Campbell B Almada AL Collins R. ISSN exercise & sport nutrition review: research & recommendations. J Int Soc Sports Nutri. (2010) 7:7. 10.1186/1550-2783-7-7 PMC6090881 30068354

[33] Ahrens HE Huettemeister J Schmidt M Kaether C von Maltzahn J. Klotho expression is a prerequisite for proper muscle stem cell function and regeneration of skeletal muscle. Skelet Muscle. (2018) 8:20. 10.1186/s13395-018-0166-x 29973273 PMC6030782

[34] Sahu A Mamiya H Shinde S Cheikhi A Winter L Vo N. Age-related declines in α-Klotho drive progenitor cell mitochondrial dysfunction and impaired muscle regeneration. Nature Commun. (2018) 9:1–14. 10.1038/s41467-018-07253-3 30451844 PMC6242898

[35] Rosenberg IH. Sarcopenia: origins and clinical relevance. Clin Geriatr Med. (2011) 27:337–9. 10.1016/j.cger.2011.03.003 21824550

[36] Cao L Morley JE. Sarcopenia is recognized as an independent condition by an international classification of disease, tenth revision, clinical modification (ICD-10-CM) code. J Am Med Direct Assoc. (2016) 17:675–7. 10.1016/j.jamda.2016.06.001 27470918

[37] Morley JE Abbatecola AM Argiles JM Baracos V Bauer J Bhasin S. Sarcopenia With Limited Mobility: An International Consensus. J Am Med Direct Assoc. (2011) 12:403–9. 10.1016/j.jamda.2011.04.014 21640657 PMC5100674

[38] Bhasin S Travison TG Manini TM Patel S Pencina KM Fielding RA. Sarcopenia definition: the position statements of the sarcopenia definition and outcomes consortium. J Am Geriatr Soc. (2020) 68:1410–8. 10.1111/jgs.16372 32150289 PMC12132920

[39] Fielding RA Vellas B Evans WJ Bhasin S Morley JE Newman AB. Sarcopenia: an undiagnosed condition in older adults. current consensus definition: prevalence, etiology, and consequences. international working group on sarcopenia. J Am Med Direct Assoc. (2011) 12:249–56. 10.1016/j.jamda.2011.01.003 21527165 PMC3377163

[40] Cruz-Jentoft AJ Bahat G Bauer J Boirie Y Bruyère O Cederholm T. Sarcopenia: revised European consensus on definition and diagnosis. Age Ageing. (2018) 48:16–31. 10.1093/ageing/afy169 30312372 PMC6322506

[41] Cruz-Jentoft AJ Baeyens JP Bauer JM Boirie Y Cederholm T Landi F. Sarcopenia: European consensus on definition and diagnosisReport of the European Working Group on Sarcopenia in Older PeopleA. J. Cruz-Gentoft. (2010) 39:412–23. 10.1093/ageing/afq034 20392703 PMC2886201

[42] Studenski SA Peters KW Alley DE Cawthon PM McLean RR Harris TB. The FNIH sarcopenia project: rationale, study description, conference recommendations, and final estimates. J Gerontol A Biol Sci Med Sci. (2014) 69:547–58. 10.1093/gerona/glu010 24737557 PMC3991146

[43] Muscaritoli M Anker SD Argilés J Aversa Z Bauer JM Biolo G. Consensus definition of sarcopenia, cachexia and pre-cachexia: joint document elaborated by Special Interest Groups (SIG) “cachexia-anorexia in chronic wasting diseases” and "nutrition in geriatrics.” *Clin Nutr*. (2010) 29:154–9. 10.1016/j.clnu.2009.12.004 20060626

[44] Buford TW Anton SD Judge AR Marzetti E Wohlgemuth SE Carter CS. Models of accelerated sarcopenia: critical pieces for solving the puzzle of age-related muscle atrophy. Ageing research reviews. (2010) 9:369–83. 10.1016/j.arr.2010.04.004 20438881 PMC3788572

[45] Keller K. Sarcopenia. Wiener Medizinische Wochenschrift. (2019) 169:157–72. 10.1007/s10354-018-0618-2 29411194

[46] Foldvari M Clark M Laviolette LC Bernstein MA Kaliton D Castaneda C. Association of muscle power with functional status in community-dwelling elderly women. J Gerontol Series A: Biol Sci Med Sci. (2000) 55:M192–M9. 10.1093/gerona/55.4.M192 10811148

[47] Gueugneau M Coudy-Gandilhon C Théron L Meunier B Barboiron C Combaret L. Skeletal muscle lipid content and oxidative activity in relation to muscle fiber type in aging and metabolic syndrome. J Gerontol Series A: Biol Sci Med Sci. (2015) 70:566–76. 10.1093/gerona/glu086 24939997

[48] Overend T Cunningham D Paterson D Lefcoe M. Thigh composition in young and elderly men determined by computed tomography. Clinical Physiol. (1992) 12:629–40. 10.1111/j.1475-097X.1992.tb00366.x 1424481

[49] Goodpaster BH Chomentowski P Ward BK Rossi A Glynn NW Delmonico MJ. Effects of physical activity on strength and skeletal muscle fat infiltration in older adults: a randomized controlled trial. J Appl Physiol. (2008) 105:1498–503. 10.1152/japplphysiol.90425.2008 18818386 PMC2584841

[50] Visser M Goodpaster BH Kritchevsky SB Newman AB Nevitt M Rubin SM. Muscle mass, muscle strength, and muscle fat infiltration as predictors of incident mobility limitations in well-functioning older persons. J Gerontol Series A: Biol Sci Med Sci. (2005) 60:324–33. 10.1093/gerona/60.3.324 15860469

[51] Hilton TN Tuttle LJ Bohnert KL Mueller MJ Sinacore DR. Excessive adipose tissue infiltration in skeletal muscle in individuals with obesity, diabetes mellitus, and peripheral neuropathy: association with performance and function. Physic Therapy. (2008) 88:1336–44. 10.2522/ptj.20080079 18801853 PMC2579904

[52] Barberi L Scicchitano BM De Rossi M Bigot A Duguez S Wielgosik A. Age-dependent alteration in muscle regeneration: the critical role of tissue niche. Biogerontology. (2013) 14:273–92. 10.1007/s10522-013-9429-4 23666344 PMC3719007

[53] Kanasaki K Kitada M Koya D. Pathophysiology of the aging kidney and therapeutic interventions. Hypertens Res. (2012) 35:1121–8. 10.1038/hr.2012.159 23076406

[54] Stenvinkel P Larsson TE. Chronic kidney disease: a clinical model of premature aging. Am J Kidney Dis. (2013) 62:339–51. 10.1053/j.ajkd.2012.11.051 23357108

[55] Kooman JP Kotanko P Schols AM Shiels PG Stenvinkel P. Chronic kidney disease and premature ageing. Nat Rev Nephrol. (2014) 10:732–42. 10.1038/nrneph.2014.185 25287433

[56] Sabatino A Regolisti G Delsante M Di Motta T Cantarelli C Pioli S. Noninvasive evaluation of muscle mass by ultrasonography of quadriceps femoris muscle in End-Stage Renal Disease patients on hemodialysis. Clinic Nutri. (2019) 38:1232–9. 10.1016/j.clnu.2018.05.004 29866494

[57] Foley RN Wang C Ishani A Collins AJ Murray AM. Kidney function and sarcopenia in the United States general population: NHANES III. Am J Nephrol. (2007) 27:279–86. 10.1159/000101827 17440263

[58] Giglio J Kamimura MA Lamarca F Rodrigues J Santin F Avesani CM. Association of sarcopenia with nutritional parameters, quality of life, hospitalization, and mortality rates of elderly patients on hemodialysis. J Renal Nutri. (2018) 28:197–207. 10.1053/j.jrn.2017.12.003 29673501

[59] Pedone C Corsonello A Bandinelli S Pizzarelli F Ferrucci L Incalzi RA. Relationship between renal function and functional decline: role of the estimating equation. J Am Med Direct Assoc. (2012) 13:84. e11-84. e14. 10.1016/j.jamda.2011.01.009 21450248 PMC5077145

[60] Pagels AA Söderkvist BK Medin C Hylander B Heiwe S. Health-related quality of life in different stages of chronic kidney disease and at initiation of dialysis treatment. Health Qual Life Outcomes. (2012) 10:1–11. 10.1186/1477-7525-10-71 22710013 PMC3511211

[61] Isoyama N Qureshi AR Avesani CM Lindholm B Bàràny P Heimbürger O. Comparative associations of muscle mass and muscle strength with mortality in dialysis patients. Clinic J Am Soc Nephrol. (2014) 9:1720–8. 10.2215/CJN.10261013 25074839 PMC4186520

[62] Sabatino A Cuppari L Stenvinkel P Lindholm B Avesani CM. Sarcopenia in chronic kidney disease: what have we learned so far? J Nephrol. (2021) 34:1347–72. 10.1007/s40620-020-00840-y 32876940 PMC8357704

[63] Reese PP Cappola AR Shults J Townsend RR Gadegbeku CA Anderson C. Physical performance and frailty in chronic kidney disease. Am J Nephrol. (2013) 38:307–15. 10.1159/000355568 24107579 PMC4019506

[64] Lattanzio F Corsonello A Abbatecola AM Volpato S Pedone C Pranno L. Relationship between renal function and physical performance in elderly hospitalized patients. Rejuvenation Res. (2012) 15:545–52. 10.1089/rej.2012.1329 22950422 PMC3549205

[65] Hartmann EL Kitzman D Rocco M Leng X Klepin H Gordon M. Physical function in older candidates for renal transplantation: an impaired population. Clinic J Am Soc Nephrol. (2009) 4:588–94. 10.2215/CJN.03860808 19261824 PMC2653669

[66] Shimamura Y Hamada K Inoue K Ogata K Ishihara M Kagawa T. Serum levels of soluble secreted α-Klotho are decreased in the early stages of chronic kidney disease, making it a probable novel biomarker for early diagnosis. Clinic Experiment Nephrol. (2012) 16:722–9. 10.1007/s10157-012-0621-7 22457086

[67] Pavik I Jaeger P Ebner L Wagner CA Petzold K Spichtig D. Secreted Klotho and FGF23 in chronic kidney disease Stage 1 to 5: a sequence suggested from a cross-sectional study. Nephrol Dial Transplant. (2012) 28:352–9. 10.1093/ndt/gfs460 23129826

[68] Koyama D Sato Y Aizawa M Maki T Kurosawa M Kuro-o M. Soluble αKlotho as a candidate for the biomarker of aging. Biochem Biophys Res Commun. (2015) 467:1019–25. 10.1016/j.bbrc.2015.10.018 26462468

[69] Matsumura Y Aizawa H Shiraki-Iida T Nagai R Kuro-o M Nabeshima Y-i. Identification of the humanklothogene and its two transcripts encoding membrane and secretedklothoprotein. Biochem Biophys Res Commun. (1998) 242:626–30. 10.1006/bbrc.1997.8019 9464267

[70] Imura A Iwano A Tohyama O Tsuji Y Nozaki K Hashimoto N. Secreted Klotho protein in sera and CSF: implication for post-translational cleavage in release of Klotho protein from cell membrane. FEBS Lett. (2004) 565:143–7. 10.1016/j.febslet.2004.03.090 15135068

[71] Chen G Liu Y Goetz R Fu L Jayaraman S Hu M-C. α-Klotho is a non-enzymatic molecular scaffold for FGF23 hormone signalling. Nature (2018) 553:461–6. 10.1038/nature25451 29342138 PMC6007875

[72] Chen C-D Podvin S Gillespie E Leeman SE Abraham CR. Insulin stimulates the cleavage and release of the extracellular domain of Klotho by ADAM10 and ADAM17. Proceed Nat Acad Sci. (2007) 104:19796–801. 10.1073/pnas.0709805104 18056631 PMC2148378

[73] Hu M-C Moe OW. Klotho as a potential biomarker and therapy for acute kidney injury. Nat Rev Nephrol. (2012) 8:423. 10.1038/nrneph.2012.92 22664739 PMC3752896

[74] Drüeke TB Massy ZA. Circulating Klotho levels: clinical relevance and relationship with tissue Klotho expression. Kidney Int. (2013) 83:13–5. 10.1038/ki.2012.370 23271484

[75] Hu MC Shi M Zhang J Addo T Cho HJ Barker SL. Renal production, uptake, and handling of circulating αKlotho. J Am Soc Nephrol. (2016) 27:79–90. 10.1681/ASN.2014101030 25977312 PMC4696570

[76] Lindberg K Amin R Moe OW Hu M-C Erben RG Wernerson AÖ. The kidney is the principal organ mediating klotho effects. J Am Soc Nephrol. (2014) 25:2169–75. 10.1681/ASN.2013111209 24854271 PMC4178446

[77] Kakareko K Rydzewska-Rosolowska A Brzosko S Gozdzikiewicz-Lapinska J Koc-Zorawska E Samocik P. The effect of nephrectomy on Klotho, FGF-23 and bone metabolism. Int Urol Nephrol. (2017) 49:681–8. 10.1007/s11255-017-1519-9 28130714 PMC5357491

[78] Thongprayoon C Neyra JA Hansrivijit P Medaura J Leeaphorn N Davis PW. Serum klotho in living kidney donors and kidney transplant recipients: a meta-analysis. J Clinic Med. (2020) 9:1834. 10.3390/jcm9061834 32545510 PMC7355868

[79] Lim K Groen A Molostvov G Lu T Lilley KS Snead D. alpha-Klotho Expression in Human Tissues. J Clin Endocrinol Metab. (2015) 100:E1308–18. 10.1210/jc.2015-1800 26280509 PMC4596032

[80] Olauson H Mencke R Hillebrands J-L Larsson TE. Tissue expression and source of circulating αKlotho. Bone. (2017) 100:19–35. 10.1016/j.bone.2017.03.043 28323144

[81] Avin KG Coen PM Huang W Stolz DB Sowa GA Dube JJ. Skeletal muscle as a regulator of the longevity protein, Klotho. Front Physiol. (2014) 5:189. 10.3389/fphys.2014.00189 24987372 PMC4060456

[82] Welc SS Wehling-Henricks M Kuro OM Thomas KA Tidball JG. Modulation of Klotho expression in injured muscle perturbs Wnt signalling and influences the rate of muscle growth. Exp Physiol. (2020) 105:132–47. 10.1113/EP088142 31724771 PMC6938556

[83] Kurosu H Ogawa Y Miyoshi M Yamamoto M Nandi A Rosenblatt KP. Regulation of fibroblast growth factor-23 signaling by klotho. J Biol Chemistr. (2006) 281:6120–3. 10.1074/jbc.C500457200 16436388 PMC2637204

[84] Shimada T Hasegawa H Yamazaki Y Muto T Hino R Takeuchi Y. FGF-23 is a potent regulator of vitamin D metabolism and phosphate homeostasis. J Bone Min Res. (2004) 19:429–35. 10.1359/JBMR.0301264 15040831

[85] Shimada T Kakitani M Yamazaki Y Hasegawa H Takeuchi Y Fujita T. Targeted ablation of Fgf23 demonstrates an essential physiological role of FGF23 in phosphate and vitamin D metabolism. J Clinic Investigat. (2004) 113:561–8. 10.1172/JCI200419081 14966565 PMC338262

[86] Goetz R Ohnishi M Ding X Kurosu H Wang L Akiyoshi J. Klotho coreceptors inhibit signaling by paracrine fibroblast growth factor 8 subfamily ligands. Mol Cell Biol. (2012) 32:1944–54. 10.1128/MCB.06603-11 22451487 PMC3347405

[87] Urakawa I Yamazaki Y Shimada T Iijima K Hasegawa H Okawa K. Klotho converts canonical FGF receptor into a specific receptor for FGF23. Nature. (2006) 444:770–4. 10.1038/nature05315 17086194

[88] Murer H Forster I Biber J. The sodium phosphate cotransporter family SLC34. Pflügers Archiv. (2004) 447:763–7. 10.1007/s00424-003-1072-5 12750889

[89] Jones G Prosser DE Kaufmann M. 25-Hydroxyvitamin D-24-hydroxylase (CYP24A1): its important role in the degradation of vitamin D. Archiv Biochemistr Biophy (2012) 523:9–18. 10.1016/j.abb.2011.11.003 22100522

[90] Jones G Prosser DE Kaufmann M. Cytochrome P450-mediated metabolism of vitamin D. J Lipid Res. (2014) 55:13–31. 10.1194/jlr.R031534 23564710 PMC3927478

[91] Meyer MB Benkusky NA Kaufmann M Lee SM Onal M Jones G. A kidney-specific genetic control module in mice governs endocrine regulation of the cytochrome P450 gene Cyp27b1 essential for vitamin D3 activation. J Biologic Chem. (2017) 292:17541–58. 10.1074/jbc.M117.806901 28808057 PMC5655528

[92] Rubinek T Shahmoon S Shabtay-Orbach A Ami MB Levy-Shraga Y Mazor-Aronovitch K. Klotho response to treatment with growth hormone and the role of IGF-I as a mediator. Metabolism. (2016) 65:1597–604. 10.1016/j.metabol.2016.08.004 27733247

[93] Rakugi H Matsukawa N Ishikawa K Yang J Imai M Ikushima M. Anti-oxidative effect of Klotho on endothelial cells through cAMP activation. Endocrine. (2007) 31:82–7. 10.1007/s12020-007-0016-9 17709902

[94] Imai M Ishikawa K Matsukawa N Kida I Ohta J Ikushima M. Klotho protein activates the PKC pathway in the kidney and testis and suppresses 25-hydroxyvitamin D3 1α-hydroxylase gene expression. Endocrine. (2004) 25:229–34. 10.1385/ENDO:25:3:229 15758250

[95] Ikushima M Rakugi H Ishikawa K Maekawa Y Yamamoto K Ohta J. Anti-apoptotic and anti-senescence effects of Klotho on vascular endothelial cells. Biochem Biophys Res Commun. (2006) 339:827–32. 10.1016/j.bbrc.2005.11.094 16325773

[96] Liu H Fergusson MM Castilho RM Liu J Cao L Chen J. Augmented Wnt signaling in a mammalian model of accelerated aging. Science. (2007) 317:803–6. 10.1126/science.1143578 17690294

[97] Mirza SB Ekhteiari Salmas R Fatmi MQ Durdagi S. Discovery of Klotho peptide antagonists against Wnt3 and Wnt3a target proteins using combination of protein engineering, protein–protein docking, peptide docking and molecular dynamics simulations. J Enzym Inhibit Med Chemistr. (2017) 32:84–98. 10.1080/14756366.2016.1235569 27766889 PMC6009926

[98] Zhou L Li Y Zhou D Tan RJ Liu Y. Loss of Klotho contributes to kidney injury by derepression of Wnt/β-catenin signaling. J Am Soc Nephrol. (2013) 24:771–85. 10.1681/ASN.2012080865 23559584 PMC3636797

[99] Doi S Zou Y Togao O Pastor JV John GB Wang L. Klotho inhibits transforming growth factor-β1 (TGF-β1) signaling and suppresses renal fibrosis and cancer metastasis in mice. J Biol Chem. (2011) 286:8655–65. 10.1074/jbc.M110.174037 21209102 PMC3048747

[100] Lim SW Jin L Luo K Jin J Shin YJ Hong SY. Klotho enhances FoxO3-mediated manganese superoxide dismutase expression by negatively regulating PI3K/AKT pathway during tacrolimus-induced oxidative stress. Cell Death Dis. (2017) 8:e2972–e. 10.1038/cddis.2017.365 28771227 PMC5596554

[101] Yamamoto M Clark JD Pastor JV Gurnani P Nandi A Kurosu H. Regulation of oxidative stress by the anti-aging hormone klotho. J Biol Chem. (2005) 280:38029–34. 10.1074/jbc.M509039200 16186101 PMC2515369

[102] Hui H Zhai Y Ao L Cleveland JC Jr Liu H. Klotho suppresses the inflammatory responses and ameliorates cardiac dysfunction in aging endotoxemic mice. Oncotarget. (2017) 8:15663–76. 10.18632/oncotarget.14933 28152512 PMC5362514

[103] Xie J Cha S-K An S-W Kuro-o M Birnbaumer L Huang C-L. Cardioprotection by Klotho through downregulation of TRPC6 channels in the mouse heart. Nat Commun. (2012) 3:1–11. 10.1038/ncomms2240 23212367 PMC3526952

[104] Lim K Lu T-S Molostvov G Lee C Lam F Zehnder D. Vascular Klotho deficiency potentiates the development of human artery calcification and mediates resistance to fibroblast growth factor 23. Circulation. (2012) 125:2243–55. 10.1161/CIRCULATIONAHA.111.053405 22492635

[105] Landi F Marzetti E Martone AM Bernabei R Onder G. Exercise as a remedy for sarcopenia. Curr Opin Clinic Nutri Metabol Care. (2014) 17:25–31. 10.1097/MCO.0000000000000018 24310054

[106] Hirai K Ookawara S Morishita Y. Sarcopenia and physical inactivity in patients with chronic kidney disease. Nephro-urol Month. (2016) 8:3. 10.5812/numonthly.37443 27570755 PMC4983408

[107] Watson KB Carlson SA Gunn JP Galuska DA O'Connor A Greenlund KJ. Physical inactivity among adults aged 50 years and older—United States, 2014. Morbid Mortal Week Rep. (2016) 65:954–8. 10.15585/mmwr.mm6536a3 27632143

[108] Manfredini F Mallamaci F Catizone L Zoccali C. The burden of physical inactivity in chronic kidney disease: is there an exit strategy? Nephrol Dial Transplant. (2012) 27:2143–5. 10.1093/ndt/gfs120 22553371

[109] Storer TW Basaria S Traustadottir T Harman SM Pencina K Li Z. Effects of testosterone supplementation for 3 years on muscle performance and physical function in older men. J Clinic Endocrinol Metabol. (2017) 102:583–93. 10.1210/jc.2016-2771 27754805 PMC5413164

[110] Wang C Cunningham G Dobs A Iranmanesh A Matsumoto AM Snyder PJ. Long-term testosterone gel (androgel) treatment maintains beneficial effects on sexual function and mood, lean and fat mass, and bone mineral density in hypogonadal men. J Clinic Endocrinol Metabol. (2004) 89:2085–98. 10.1210/jc.2003-032006 15126525

[111] Snyder PJ Peachey H Berlin JA Hannoush P Haddad G Dlewati A. Effects of testosterone replacement in hypogonadal men. J Clin Endocrinol Metab. (2000) 85:2670–7. 10.1210/jc.85.8.2670 10946864

[112] Papadakis MA Grady D Black D Tierney MJ Gooding GA Schambelan M. Growth hormone replacement in healthy older men improves body composition but not functional ability. Ann Intern Med. (1996) 124:708–16. 10.7326/0003-4819-124-8-199604150-00002 8633830

[113] Becker C Lord SR Studenski SA Warden SJ Fielding RA Recknor CP. Myostatin antibody (LY2495655) in older weak fallers: a proof-of-concept, randomised, phase 2 trial. Lancet Diabet Endocrinol. (2015) 3:948–57. 10.1016/S2213-8587(15)00298-3 26516121

[114] Rooks DS Laurent D Praestgaard J Rasmussen S Bartlett M Tankó LB. Effect of bimagrumab on thigh muscle volume and composition in men with casting-induced atrophy. J Cachexia Sarcopenia Muscle. (2017) 8:727–34. 10.1002/jcsm.12205 28905498 PMC5659065

[115] Massimino E Izzo A Riccardi G Della Pepa G. The impact of glucose-lowering drugs on Sarcopenia in Type 2 diabetes: current evidence and underlying mechanisms. Cells. (2021) 10:1958. 10.3390/cells10081958 34440727 PMC8393336

[116] Sumukadas D Witham MD Struthers AD McMurdo ME. Effect of perindopril on physical function in elderly people with functional impairment: a randomized controlled trial. Cmaj. (2007) 177:867–74. 10.1503/cmaj.061339 17923654 PMC1995143

[117] Garcia JM Boccia RV Graham CD Yan Y Duus EM Allen S. Anamorelin for patients with cancer cachexia: an integrated analysis of two phase 2, randomised, placebo-controlled, double-blind trials. Lancet Oncol. (2015) 16:108–16. 10.1016/S1470-2045(14)71154-4 25524795

[118] Brown LA Guzman SD Brooks SV. Emerging molecular mediators and targets for age-related skeletal muscle atrophy. Transl Res. (2020) 221:44–57. 10.1016/j.trsl.2020.03.001 32243876 PMC8026108

[119] Zhao Y Zhao M-M Cai Y Zheng M-F Sun W-L Zhang S-Y. Mammalian target of rapamycin signaling inhibition ameliorates vascular calcification *via* Klotho upregulation. Kidney Int. (2015) 88:711–21. 10.1038/ki.2015.160 26061549

[120] de Cavanagh EM Inserra F Ferder L. Angiotensin II blockade: how its molecular targets may signal to mitochondria and slow aging. coincidences with calorie restriction and mtor inhibition. Am J Physiol-Heart Circulat Physiol. (2015) 309:H15–H44. 10.1152/ajpheart.00459.2014 25934099

[121] Hsu S-C Huang S-M Lin S-H Ka S-M Chen A Shih M-F. Testosterone increases renal anti-aging klotho gene expression *via* the androgen receptor-mediated pathway. Biochem J. (2014) 464:221–9. 10.1042/BJ20140739 25163025

[122] Amaro-Gahete FJ Jurado-Fasoli L Gutiérrez Á Ruiz JR Castillo MJ. Association of physical activity and fitness with S-Klotho plasma levels in middle-aged sedentary adults: The FIT-AGEING study. Maturitas. (2019) 123:25–31. 10.1016/j.maturitas.2019.02.001 31027673

[123] Amaro-Gahete FJ De-la-O A Jurado-Fasoli L Espuch-Oliver A de Haro T Gutierrez A. Body composition and S-Klotho plasma levels in middle-aged adults: A cross-sectional study. Rejuvenat Res. (2019) 22:478–83. 10.1089/rej.2018.2092 30672377

[124] Valenzuela PL Cobo F Diez-Vega I Sánchez-Hernández R Pedrero-Chamizo R Verde-Rello Z. Physical performance, plasma S-klotho, and all-cause mortality in elderly dialysis patients: A prospective cohort study. Experiment Gerontol. (2019) 122:123–8. 10.1016/j.exger.2019.05.003 31077742

[125] Baldan A Giusti A Bosi C Malaventura C Musso M Forni GL. Klotho, a new marker for osteoporosis and muscle strength in β-thalassemia major. Blood Cells Mol Dis. (2015) 55:396–401. 10.1016/j.bcmd.2015.08.004 26460265

[126] Chalhoub D Marques E Meirelles O Semba RD Ferrucci L Satterfield S. Association of serum klotho with loss of bone mineral density and fracture risk in older adults. J Am Geriatrics Soc. (2016) 64:e304–e8. 10.1111/jgs.14661 27910102 PMC5173411

[127] Dote-Montero M Amaro-Gahete FJ Jurado-Fasoli L Gutierrez A Castillo MJ. Study of the association of DHEAS, testosterone and cortisol with S-Klotho plasma levels in healthy sedentary middle-aged adults. Experiment Gerontol. (2019) 121:55–61. 10.1016/j.exger.2019.03.010 30928678

[128] Fukasawa H Ishigaki S Kinoshita-Katahashi N Niwa H Yasuda H Kumagai H. Plasma levels of fibroblast growth factor-23 are associated with muscle mass in haemodialysis patients. Nephrology. (2014) 19:784–90. 10.1111/nep.12333 25185859

[129] Matsubara T Miyaki A Akazawa N Choi Y Ra S-G Tanahashi K. Aerobic exercise training increases plasma Klotho levels and reduces arterial stiffness in postmenopausal women. Am J Physiol Heart Circulat Physiol. (2014) 306:H348–H55. 10.1152/ajpheart.00429.2013 24322608

[130] Mostafidi E Moeen A Nasri H Hagjo AG Ardalan M. Serum Klotho levels in trained athletes. Nephro-Urology Monthly. (2016) 8. 10.5812/numonthly.30245 26981496 PMC4780197

[131] Patel M Donaldson A Lewis A Natanek S Lee J Andersson Y. Klotho and smoking–an interplay influencing the skeletal muscle function deficits that occur in COPD. Respirat Med. (2016) 113:50–6. 10.1016/j.rmed.2016.02.004 27021580

[132] Polat Y Yalcin A Yazihan N Bahsi R Surmeli DM Akdas S. The relationship between frailty and serum alpha klotho levels in geriatric patients. Archiv Gerontol Geriatr. (2020) 91:104225. 10.1016/j.archger.2020.104225 32905907

[133] Rosa TS Neves RVP Deus LA Sousa CV da Silva Aguiar S de Souza MK. Sprint and endurance training in relation to redox balance, inflammatory status and biomarkers of aging in master athletes. Nitric Oxide. (2020) 102:42–51. 10.1016/j.niox.2020.05.004 32565116

[134] Sanz B Arrieta H Rezola-Pardo C Fernández-Atutxa A Garin-Balerdi J Arizaga N. Low serum klotho concentration is associated with worse cognition, psychological components of frailty, dependence, and falls in nursing home residents. Scientific Rep. (2021) 11:9098. 10.1038/s41598-021-88455-6 33907242 PMC8079365

[135] Le Grand F Rudnicki MA. Skeletal muscle satellite cells and adult myogenesis. Curr Opin Cell Biol. (2007) 19:628–33. 10.1016/j.ceb.2007.09.012 17996437 PMC2215059

[136] Tidball JG Villalta SA. Regulatory interactions between muscle and the immune system during muscle regeneration. Am J Physiol Regulat Integrat Comparat Physiol. (2010) 298:R1173–R87. 10.1152/ajpregu.00735.2009 20219869 PMC2867520

[137] Mahdy MA. Skeletal muscle fibrosis: an overview. Cell Tissue Res. (2019) 375:575–88. 10.1007/s00441-018-2955-2 30421315

[138] Shefer G Van de Mark DP Richardson JB Yablonka-Reuveni Z. Satellite-cell pool size does matter: defining the myogenic potency of aging skeletal muscle. Development Biol. (2006) 294:50–66. 10.1016/j.ydbio.2006.02.022 16554047 PMC2710453

[139] Avin KG Chen NX Organ JM Zarse C O'Neill K Conway RG. Skeletal muscle regeneration and oxidative stress are altered in chronic kidney disease. PLoS ONE. (2016) 11:e0159411. 10.1371/journal.pone.0159411 27486747 PMC4972446

[140] Wehling-Henricks M Li Z Lindsey C Wang Y Welc SS Ramos JN. Klotho gene silencing promotes pathology in the mdx mouse model of Duchenne muscular dystrophy. Hum Mol Genet. (2016) 25:2465–82. 10.1093/hmg/ddw111 27154199 PMC5181628

[141] von Maltzahn J Chang NC Bentzinger CF Rudnicki MA. Wnt signaling in myogenesis. Trends Cell Biol. (2012) 22:602–9. 10.1016/j.tcb.2012.07.008 22944199 PMC3479319

[142] Brack AS Conboy MJ Roy S Lee M Kuo CJ Keller C. Increased Wnt signaling during aging alters muscle stem cell fate and increases fibrosis. Science. (2007) 317:807–10. 10.1126/science.1144090 17690295

[143] Zhang L Wang XH Wang H Du J Mitch WE. Satellite cell dysfunction and impaired IGF-1 signaling cause CKD-induced muscle atrophy. J Am Soc Nephrol. (2010) 21:419–27. 10.1681/ASN.2009060571 20056750 PMC2831855

[144] Abramowitz MK Paredes W Zhang K Brightwell CR Newsom JN Kwon H-J. Skeletal muscle fibrosis is associated with decreased muscle inflammation and weakness in patients with chronic kidney disease. Am J Physiol Renal Physiol. (2018) 315:F1658–F69. 10.1152/ajprenal.00314.2018 30280599 PMC6336993

[145] Dong J Dong Y Chen Z Mitch WE Zhang L. The pathway to muscle fibrosis depends on myostatin stimulating the differentiation of fibro/adipogenic progenitor cells in chronic kidney disease. Kidney Int. (2017) 91:119–28. 10.1016/j.kint.2016.07.029 27653838 PMC5179308

[146] Carlson ME Conboy MJ Hsu M Barchas L Jeong J Agrawal A. Relative roles of TGF-β1 and Wnt in the systemic regulation and aging of satellite cell responses. Aging cell. (2009) 8:676–89. 10.1111/j.1474-9726.2009.00517.x 19732043 PMC2783265

[147] Li Y Foster W Deasy BM Chan Y Prisk V Tang Y. Transforming growth factor-β1 induces the differentiation of myogenic cells into fibrotic cells in injured skeletal muscle: a key event in muscle fibrogenesis. Am J Pathol. (2004) 164:1007–19. 10.1016/S0002-9440(10)63188-4 14982854 PMC1614716

[148] Blasco MA. Telomere length, stem cells and aging. Nat Chem Biol. (2007) 3:640–9. 10.1038/nchembio.2007.38 17876321

[149] Xi H Li C Ren F Zhang H Zhang L. Telomere, aging and age-related diseases. Aging Clinic Experiment Res. (2013) 25:139–46. 10.1007/s40520-013-0021-1 23739898

[150] Marzetti E Lorenzi M Antocicco M Bonassi S Celi M Mastropaolo S. Shorter telomeres in peripheral blood mononuclear cells from older persons with sarcopenia: results from an exploratory study. Front Aging Neurosci. (2014) 6:233. 10.3389/fnagi.2014.00233 25221511 PMC4147848

[151] Bernabeu-Wittel M Gómez-Díaz R González-Molina Á Vidal-Serrano S Díez-Manglano J Salgado F. Oxidative stress, telomere shortening, and apoptosis associated to sarcopenia and frailty in patients with multimorbidity. J Clinic Med. (2020) 9:2669. 10.3390/jcm9082669 32824789 PMC7464426

[152] Lorenzi M Bonassi S Lorenzi T Giovannini S Bernabei R Onder G. A review of telomere length in sarcopenia and frailty. Biogerontology. (2018) 19:209–21. 10.1007/s10522-018-9749-5 29549539

[153] Kadi F Ponsot E. The biology of satellite cells and telomeres in human skeletal muscle: effects of aging and physical activity. Scand J Med Sci Sports. (2010) 20:39–48. 10.1111/j.1600-0838.2009.00966.x 19765243

[154] Ullah M Sun Z. Klotho deficiency accelerates stem cells aging by impairing telomerase activity. J Gerontol Series A. (2018) 74:1396–407. 10.1093/gerona/gly261 30452555 PMC6696722

[155] Peterson CM Johannsen DL Ravussin E. Skeletal muscle mitochondria and aging: a review. J Aging Res. (2012) 2012:184821. 10.1155/2012/194821 22888430 PMC3408651

[156] Thome T Kumar RA Burke SK Khattri RB Salyers ZR Kelley RC. Impaired muscle mitochondrial energetics is associated with uremic metabolite accumulation in chronic kidney disease. JCI insight. (2021) 6:1. 10.1172/jci.insight.139826 33290279 PMC7821598

[157] Wagatsuma A Kotake N Yamada S. Muscle regeneration occurs to coincide with mitochondrial biogenesis. Mol Cell Biochemistr. (2011) 349:139–47. 10.1007/s11010-010-0668-2 21110070

[158] Miao J Huang J Luo C Ye H Ling X Wu Q. Klotho retards renal fibrosis through targeting mitochondrial dysfunction and cellular senescence in renal tubular cells. Physiol Rep. (2021) 9:e14696. 10.14814/phy2.14696 33463897 PMC7814487

[159] Bhattacharya D Scimè A. Mitochondrial function in muscle stem cell fates. Front Cell Development Biol. (2020) 8:480. 10.3389/fcell.2020.00480 32612995 PMC7308489

[160] Fulle S Protasi F Di Tano G Pietrangelo T Beltramin A Boncompagni S. The contribution of reactive oxygen species to sarcopenia and muscle ageing. Experiment Gerontol. (2004) 39:17–24. 10.1016/j.exger.2003.09.012 14724060

[161] Takemura K Nishi H Inagi R. Mitochondrial dysfunction in kidney disease and uremic sarcopenia. Front Physiol. (2020) 11:56503. 10.3389/fphys.2020.565023 33013483 PMC7500155

[162] Izbeki F Asuzu DT Lorincz A Bardsley MR Popko LN Choi KM. Loss of Kitlow progenitors, reduced stem cell factor and high oxidative stress underlie gastric dysfunction in progeric mice. J Physiol. (2010) 588:3101–17. 10.1113/jphysiol.2010.191023 20581042 PMC2956948

[163] Oyewole AO Birch-Machin MA. Mitochondria-targeted antioxidants. FASEB J. (2015) 29:4766–71. 10.1096/fj.15-275404 26253366

[164] Zhou J Liu B Liang C Li Y Song Y-H. Cytokine signaling in skeletal muscle wasting. Trends Endocrinol Metabol. (2016) 27:335–47. 10.1016/j.tem.2016.03.002 27025788

[165] Cai D Frantz JD Tawa NE Melendez PA Oh B-C Lidov HGW. IKKβ/NF-κB activation causes severe muscle wasting in mice. Cell. (2004) 119:285–98. 10.1016/j.cell.2004.09.027 15479644

[166] Lecker SH Goldberg AL Mitch WE. Protein degradation by the ubiquitin–proteasome pathway in normal and disease states. J Am Soc Nephrol. (2006) 17:1807–19. 10.1681/ASN.2006010083 16738015

[167] Li Y-P. TNF-α is a mitogen in skeletal muscle. Am J Physiol Cell Physiol. (2003) 285:C370–C6. 10.1152/ajpcell.00453.2002 12711593

[168] Ohyama Y Kurabayashi M Masuda H Nakamura T Aihara Y Kaname T. Molecular cloning of RatklothocDNA: markedly decreased expression ofklothoby acute inflammatory stress. Biochem Biophysic Res Commun. (1998) 251:920–5. 10.1006/bbrc.1998.9576 9791011

[169] Thurston RD Larmonier CB Majewski PM Ramalingam R Midura-Kiela M Laubitz D. Tumor necrosis factor and interferon-γ down-regulate Klotho in mice with colitis. Gastroenterology. (2010) 138:1384–94. e2. 10.1053/j.gastro.2009.12.002 20004202 PMC3454518

[170] Hu MC Shi M Zhang J Quiñones H Griffith C Kuro-o M. Klotho deficiency causes vascular calcification in chronic kidney disease. J Am Soc Nephrol. (2011) 22:124–36. 10.1681/ASN.2009121311 21115613 PMC3014041

[171] Moreno JA Izquierdo MC Sanchez-Niño MD Suárez-Alvarez B Lopez-Larrea C Jakubowski A. The inflammatory cytokines TWEAK and TNFα reduce renal klotho expression through NFκB. J Am Soc Nephrol. (2011) 22:1315–25. 10.1681/ASN.2010101073 21719790 PMC3137579

[172] Wehling-Henricks M Welc SS Samengo G Rinaldi C Lindsey C Wang Y. Macrophages escape Klotho gene silencing in the mdx mouse model of Duchenne muscular dystrophy and promote muscle growth and increase satellite cell numbers through a Klotho-mediated pathway. Hum Mol Genetics. (2018) 27:14–29. 10.1093/hmg/ddx380 29040534 PMC5886268

[173] Lim K McGregor G Coggan AR Lewis GD Moe SM. Cardiovascular functional changes in chronic kidney disease: integrative physiology, pathophysiology and applications of cardiopulmonary exercise testing. Front Physiol. (2020) 11:55. 10.3389/fphys.2020.572355 33041870 PMC7522507

[174] Albouaini K Egred M Alahmar A Wright DJ. Cardiopulmonary exercise testing and its application. Postgrad Med J. (2007) 83:675–82. 10.1136/hrt.2007.121558 17989266 PMC2734442

[175] Fleg JL Morrell CH Bos AG Brant LJ Talbot LA Wright JG. Accelerated longitudinal decline of aerobic capacity in healthy older adults. Circulation. (2005) 112:674–82. 10.1161/CIRCULATIONAHA.105.545459 16043637

[176] Maron BA Cockrill BA Waxman AB Systrom DM. The invasive cardiopulmonary exercise test. Circulation. (2013) 127:1157–64. 10.1161/CIRCULATIONAHA.112.104463 23479667

[177] Chodzko-Zajko WJ Proctor DN Fiatarone Singh MA Minson CT Nigg CR Salem GJ. Exercise and physical activity for older adults. Med Sci Sports Exer. (2009) 41:7. 10.1249/MSS.0b013e3181a0c95c 19516148

[178] Fiuza-Luces C Garatachea N Berger NA Lucia A. Exercise is the real polypill. Physiology. (2013) 19:13. 10.1152/physiol.00019.2013 23997192

[179] Santos-Dias A MacKenzie B Oliveira-Junior MC Moyses RM Consolim-Colombo FM Vieira RP. Longevity protein klotho is induced by a single bout of exercise. Br J Sports Med. (2017) 51:549–50. 10.1136/bjsports-2016-096139 27251899

[180] Tan S-J Chu MM Toussaint ND Cai MM Hewitson TD Holt SG. High-intensity physical exercise increases serum α-klotho levels in healthy volunteers. J Circulat Biomark. (2018) 7:1849454418794582. 10.1177/1849454418794582 30147756 PMC6100126

[181] Saghiv MS Sira DB Goldhammer E Sagiv M. The effects of aerobic and anaerobic exercises on circulating soluble-Klotho and IGF-I in young and elderly adults and in CAD patients. J Circ Biomark. (2017) 6:1849454417733388. 10.1177/1849454417733388 29081845 PMC5644364

[182] Rahimi S Khademvatani K Zolfaghari MR. Association of circular Klotho and insulin-like growth factor 1 with cardiac hypertrophy indexes in athlete and non-athlete women following acute and chronic exercise. Biochem Biophysic Res Commun. (2018) 505:448–52. 10.1016/j.bbrc.2018.09.138 30269819

[183] Rao Z Zheng L Huang H Feng Y Shi R. α-Klotho expression in mouse tissues following acute exhaustive exercise. Front Physiol. (2019) 10:1498. 10.3389/fphys.2019.01498 31920703 PMC6919267

[184] Iturriaga T Yvert T Sanchez-Lorente IM Diez-Vega I Fernandez-Elias VE Sanchez-Barroso L. Acute impacts of different types of exercise on circulating α-Klotho protein levels. Front Physiol. (2021) 12:716473. 10.3389/fphys.2021.716473 34539440 PMC8440965

[185] Ramez M Rajabi H Ramezani F Naderi N Darbandi-Azar A Nasirinezhad F. The greater effect of high-intensity interval training versus moderate-intensity continuous training on cardioprotection against ischemia-reperfusion injury through Klotho levels and attenuate of myocardial TRPC6 expression. BMC Cardiovascul Disord. (2019) 19:118. 10.1186/s12872-019-1090-7 31096903 PMC6524218

[186] Ramez M Ramezani F Nasirinezhad F Rajabi H. High-intensity interval training increases myocardial levels of Klotho and protects the heart against ischaemia-reperfusion injury. Exp Physiol. (2020) 105:652–65. 10.1113/EP087994 32052504

[187] Gaitán JM Moon HY Stremlau M Dubal DB Cook DB Okonkwo OC. Effects of aerobic exercise training on systemic biomarkers and cognition in late middle-aged adults at risk for Alzheimer's Disease. Front endocrinol. (2021) 12:562. 10.3389/fendo.2021.660181 34093436 PMC8173166

[188] Saghiv MS Ben-Sira D Goldhammer E Sagiv M. Are S-Klotho's Maximal concentrations dependent on exercise intensity and time in young adult males? (2019).

[189] Saghiv M Sherve C Ben-Sira D Sagiv M Goldhammer E. Aerobic training effect on blood S-Klotho levels in coronary artery disease patients. J Clinic Experiment Cardiol. (2016) 7:1–4. 10.4172/2155-9880.1000464

[190] Saghiv MS Goldhammer E Radzishevski E. The impact of 12 weeks exercise training on circulating soluble-klotho and Pro-BNP in coronary artery disease patients. J Cardiol Vasc Res. (2017) 1:1–4. 10.33425/2639-8486.1005

[191] Ji N Luan J Hu F Zhao Y Lv B Wang W. Aerobic exercise-stimulated Klotho upregulation extends life span by attenuating the excess production of reactive oxygen species in the brain and kidney. Experiment Therapeutic Med. (2018) 16:3511–7. 10.3892/etm.2018.6597 30233703 PMC6143843

[192] Dalise S Cavalli L Ghuman H Wahlberg B Gerwig M Chisari C. Biological effects of dosing aerobic exercise and neuromuscular electrical stimulation in rats. Scientific Rep. (2017) 7:1–13. 10.1038/s41598-017-11260-7 28883534 PMC5589775

[193] Amaro-Gahete FJ De-la-O A Jurado-Fasoli L Espuch-Oliver A de Haro T Gutierrez A. Exercise training increases the S-Klotho plasma levels in sedentary middle-aged adults: a randomised controlled trial. the FIT-AGEING study. J Sports Sci. (2019) 37:2175–83. 10.1080/02640414.2019.1626048 31164040

[194] Middelbeek RJW Motiani P Brandt N Nigro P Zheng J Virtanen KA. Exercise intensity regulates cytokine and klotho responses in men. Nutri Diabet. (2021) 11:5. 10.1038/s41387-020-00144-x 33414377 PMC7791135

[195] Fakhrpour R Khosroshahi HT Ebrahim K Ahmadizad S Abbasnejad M Abbasi MM. Effect of sixteen weeks combined training on FGF-23, Klotho, and Fetuin-A levels in patients on maintenance hemodialysis. Iranian J Kid Dis. (2020) 14:212–8. Available online at: http://www.ijkd.org/index.php/ijkd/article/view/4886 32361698

[196] Neves RV Corrêa HL Deus LA Reis AL Souza MK Simoes HG. Dynamic not isometric training blunts osteo-renal disease and improves the sclerostin/FGF23/Klotho axis in maintenance hemodialysis patients: a randomized clinical trial. J Appl Physiol. (2021) 130:508–16. 10.1152/japplphysiol.00416.2020 33242299

[197] Neyra JA Hu MC Moe OW. Klotho in clinical nephrology: diagnostic and therapeutic implications. Clinic J Am Soc Nephrol. (2021) 16:162–76. 10.2215/CJN.02840320 32699047 PMC7792642

[198] Lau WL Leaf EM Hu MC Takeno MM Kuro-o M Moe OW. Vitamin D receptor agonists increase klotho and osteopontin while decreasing aortic calcification in mice with chronic kidney disease fed a high phosphate diet. Kidney Int. (2012) 82:1261–70. 10.1038/ki.2012.322 22932118 PMC3511664

[199] Abraham CR Chen C Cuny GD Glicksman MA Zeldich E. Small-molecule Klotho enhancers as novel treatment of neurodegeneration. Future Med Chem. (2012) 4:1671-9. 10.4155/fmc.12.134 22924505 PMC3564652

[200] Hu M-C Shi M Zhang J Quiñones H Kuro-o M Moe OW. Klotho deficiency is an early biomarker of renal ischemia–reperfusion injury and its replacement is protective. Kidney Int. (2010) 78:1240–51. 10.1038/ki.2010.328 20861825 PMC3237296

[201] Wang Y Sun Z. Klotho gene delivery prevents the progression of spontaneous hypertension and renal damage. Hypertension. (2009) 54:810–7. 10.1161/HYPERTENSIONAHA.109.134320 19635988 PMC2814175

[202] Saito Y Nakamura T Ohyama Y Suzuki T Iida A Shiraki-Iida T. In vivo klotho gene delivery protects against endothelial dysfunction in multiple risk factor syndrome. Biochem Biophysic Res Commun. (2000) 276:767–72. 10.1006/bbrc.2000.3470 11027545

[203] Xie J Yoon J An S-W Kuro-o M Huang C-L. Soluble klotho protects against uremic cardiomyopathy independently of fibroblast growth factor 23 and phosphate. J Am Soc of Nephrol. (2015) 26:1150–60. 10.1681/ASN.2014040325 25475745 PMC4413766

[204] Cheikhi A Barchowsky A Sahu A Shinde SN Pius A Clemens ZJ. Klotho: an elephant in aging research. J Gerontol Series A. (2019) 74:1031–42. 10.1093/gerona/glz061 30843026 PMC7330474

[205] Dalton GD Xie J An S-W Huang C-L. New Insights into the Mechanism of Action of Soluble Klotho. Front Endocrinol. (2017) 8:323. 10.3389/fendo.2017.00323 29250031 PMC5715364

[206] Fanzani A Zanola A Faggi F Papini N Venerando B Tettamanti G. Implications for the mammalian sialidases in the physiopathology of skeletal muscle. Skeletal Muscle. (2012) 2:23. 10.1186/2044-5040-2-23 23114189 PMC3534598

